# Electric field and SAR reduction in high-impedance RF arrays by using high permittivity materials for 7T MR imaging

**DOI:** 10.1371/journal.pone.0305464

**Published:** 2024-07-03

**Authors:** Aditya A. Bhosale, Yunkun Zhao, Xiaoliang Zhang

**Affiliations:** 1 Department of Biomedical Engineering, State University of New York at Buffalo, Buffalo, NY, United States of America; 2 Department of Electrical Engineering, State University of New York at Buffalo, Buffalo, NY, United States of America; King’s College London, UNITED KINGDOM

## Abstract

In the field of ultra-high field MR imaging, the challenges associated with higher frequencies and shorter wavelengths necessitate rigorous attention to multichannel array design. While the need for such arrays remains, and efforts to increase channel counts continue, a persistent impediment—inter-element coupling—constantly hinders development. This coupling degrades current and field distribution, introduces noise correlation between channels, and alters the frequency of array elements, affecting image quality and overall performance. The goal of optimizing ultra-high field MRI goes beyond resolving inter-element coupling and includes significant safety considerations related to the design changes required to achieve high-impedance coils. Although these coils provide excellent isolation, the higher impedance needs special design changes. However, such changes pose a significant safety risk in the form of strong electric fields across low-capacitance lumped components. This process may raise Specific Absorption Rate (SAR) values in the imaging subject, increasing power deposition and, as a result, the risk of tissue heating-related injury. To balance the requirement of inter-element decoupling with the critical need for safety, we suggest a new solution. Our method uses high-dielectric materials to efficiently reduce electric fields and SAR values in the imaging sample. This intervention tries to maintain B1 efficiency and inter-element decoupling within the existing array design, which includes high-impedance coils. Our method aims to promote the full potential of ultra-high field MRI by alleviating this critical safety concern with minimal changes to the existing array setup.

## Introduction

Multi-channel arrays outweigh volume coils in terms of improved signal-to-noise ratio and faster acquisition when used in combination with parallel imaging techniques [1–4]. At ultra-high-field magnetic resonance imaging (7T and above), the Larmor frequency increases and the wavelength decreases, making it challenging to develop larger imaging coils, such as volume coils [5–8]. The addition of the channel count in a multi-channel array is regarded as advantageous due to the positive impact of higher channel numbers on the signal-to-noise ratio (SNR), consequently leading to an enhancement in the magnetic resonance (MR) image, but more channels also present a technical challenge known as inter-element coupling [9–13]. Inter-element coupling impacts the current and B1 field distribution and contributes to correlated noise that degrades SNR and parallel imaging performance [14–16]. It is critical to keep inter-element coupling to a minimum to preserve imaging performance. Numerous efforts have been made to reduce inter-element coupling between array elements while maintaining the present element count. One well-known example is overlapping neighboring elements in arrays to minimize interaction between the surrounding components [17, 18]. In receive-only arrays, this method is generally used together with a low input impedance pre-amplifier decoupling method to reduce interaction between non-adjacent elements although it is not readily feasible for transceiver arrays [9, 18]. Capacitive/inductive decoupling networks constitute additional methods for improving inter-element isolation between array elements [19–21]. Other attempts have been made to reduce coupling by utilizing the metamaterial substrate to reduce or eliminate the induced currents [22–27]. All of the aforementioned approaches provide excellent inter-element isolation, which improves imaging performance but also increases the complexity of array designs due to the added decoupling circuitry. The high-impedance coil design is another decoupling solution that has been revisited and further investigated recently [18, 28–33]. This method not only improves inter-element isolation but also simplifies array construction by eliminating the need for additional circuitry, leading to a robust array design method with high efficiency and durability, particularly for flexible multichannel arrays.

The high-impedance coils are defined as coils specifically designed to increase impedance, and various approaches can be employed to achieve this objective. One notable method involves the use of fabrication techniques, where coaxial cables are used to attain the desired high impedance. Another noteworthy design, known as the self-decoupled coil, relies on a conventional LC loop with intentional placement of a low-value capacitor opposite to the feed port to achieve high impedance and, consequently, enhanced inter-element isolation. To introduce high impedance into the resonator circuit, small values of lumped capacitors are often selected. High electric fields are created across these low-capacitance capacitors. Because the specific absorption rate (SAR) is proportional to the square of the electric fields, higher electric fields imply higher SAR values [34–36]. SAR is a measure of how much power an RF field deposits in a certain mass of tissue and is a key cause of tissue heating in MR imaging. As a result, keeping SAR values within a specific range and particularly FDA guidelines is crucial to ensuring human safety during MR imaging tests. High-impedance coils preserve the geometry of the conventional loop while offering excellent inter-element isolation between array elements. However, the high impedance property may result in high electric field generation over the lumped elements with low capacitance, resulting in high E-spots on the coil and higher power being deposited into the tissue, causing tissue heating or burns, a known safety hazard that must be addressed properly. To address this issue, we propose to utilize the known potential of the high dielectric constant material [37–40]. Prior studies have explored the use of high dielectric materials to positively alter B1 field distributions and integrate them into coil fabrication, as these materials can affect the resonant frequency and absorb E-fields. Additional research has investigated the utilization of materials with high dielectric constant (HDC) as resonators [41–45], as well as prioritizing the improvement of safety in ultra-high field strength MRI [46–48]. This work focuses on enhancing the safety considerations related to high-impedance coil array designs in UHF MRI while maintaining their electromagnetic decoupling performance. We present a novel application of HDC materials to reduce electric fields and Specific Absorption Rate (SAR) values in high-impedance coil arrays.

In this work, we propose the use of a thin, high-dielectric sheet to mitigate the potential dangers of tissue heating or burns caused by the high electric field produced by the low-capacitance lumped elements presented in these lumped-element high-impedance coil arrays. To demonstrate our methodology, we propose utilizing a well-known instance of a coil with high impedance, namely the self-decoupled coil, to exemplify the application of high dielectric materials with a focus on safety. It is important to emphasize that our suggested approach is flexible and may be utilized for a wide array of high-impedance coil designs. This provides a versatile solution to address safety concerns in different coil configurations.

To conclude our investigation, we used rigorous numerical simulation analysis to assess scattering parameters, electric fields, Specific Absorption Rate (SAR), and B1 fields across a range of human tissue properties in controlled experimental scenarios. These scenarios included changes in the size and relative permittivity values of the high dielectric constant material sheet, as well as adjustments to its distance from high-impedance coils. In addition, we built a prototype and ran extensive bench tests to validate our proposed methodology. The results of these experiments not only validate but also demonstrate, the practicality of our concept. This comprehensive approach, which combines numerical simulations and real-world experimentation, strengthens and validates the proposed safety-centric methodology for high-impedance coil designs in ultra-high-field MRI applications.

## Methods

This section goes over the several cases we investigated for our proposed technique. A high-impedance coil without any high dielectric constant material is looked at and compared to an instance with high dielectric constant material placed between the phantom and the high-impedance coil. In our initial experiments, we investigated the use of a small piece of high dielectric constant (HDC) material strategically placed to cover the low-value lumped element of the high-impedance coil. The goal was to reduce electric fields by allowing a small HDC material piece to absorb a significant amount of electromagnetic energy. However, these early experiments revealed limitations, as a significant amount of electric fields passed through the small HDC material piece, resulting in insufficient reduction of electric fields on the phantom surface. Following this observation, we conducted additional experiments to improve the method’s effectiveness by increasing the area covered by the HDC material. This iterative process allowed us to establish a relationship between the area of HDC materials and their ability to reduce electric fields. Notably, our findings showed that HDC materials with a larger coverage area were more effective in achieving the desired reduction in electric fields. Building on this insight, we increased the size of the HDC material to completely cover the high-impedance coil. This change aimed to maximize the positive effects seen in our initial experiments with partial placement. The increased coverage area of the HDC material resulted in a more significant reduction in electric fields, which was a valuable optimization for our proposed technique.

To verify that the coil was tuned at 300 MHz and precisely matched at 50 ohms, the relative permittivity, and distance of the high dielectric constant material from the coil were varied within a specific range. A cylindrical phantom with a 30 cm diameter and 30 cm height was used to evaluate the inter-element isolation, electric field, B1+ fields, and SAR values for each case. The phantom was assigned different tissue parameters, such as the human brain, breast fat, kidney, and tendon/ligament, to evaluate all the resultant parameters in depth. In addition to phantom evaluations, the study included comprehensive assessments such as bench testing and the corresponding identical simulation evaluation. The numerical voxel simulation was carried out using an 8-channel array configuration, with and without the HDC material. This configuration was specifically designed for human brain imaging, allowing for a thorough investigation into how the HDC material affects the coil’s performance and imaging capabilities.

### A. High-impedance coils without the high dielectric constant material

The inter-element isolation of two 10 × 10cm^2^ high-impedance coil resonators was evaluated. The 1 cm distance between the two resonators was maintained. A cylindrical phantom with a diameter of 30 cm and a height of 30 cm was used and assigned the properties of human brain tissue (conductivity *σ* = 0.6 S/m and permittivity *ε*_*r*_ = 50). The same 1.5 cm distance was maintained between the cylindrical phantom and the resonators. By selecting the appropriate capacitors and inductors, the high-impedance coils were tuned to 300 MHz, and their impedances were matched to 50 ohms. The elements had a low-value Cmode capacitor (0.35 pF) placed opposite the feed port, which allowed the resonators to attain a high impedance and create a dipole-like open-path current pattern for exceptional decoupling behavior. Schematic co-simulation from Dassault Systemes’ CST studio suite was used to precisely select the low-value Cmode capacitor and Xarm lumped inductor (40 nH) to tune the coil at 300 MHz and achieve the self-decoupling property of the high-impedance coil. In addition, a shunt Cmatch capacitor (15 pF) was connected to the input port to match the impedance of each channel to 50 ohms.

**Fig 1:**
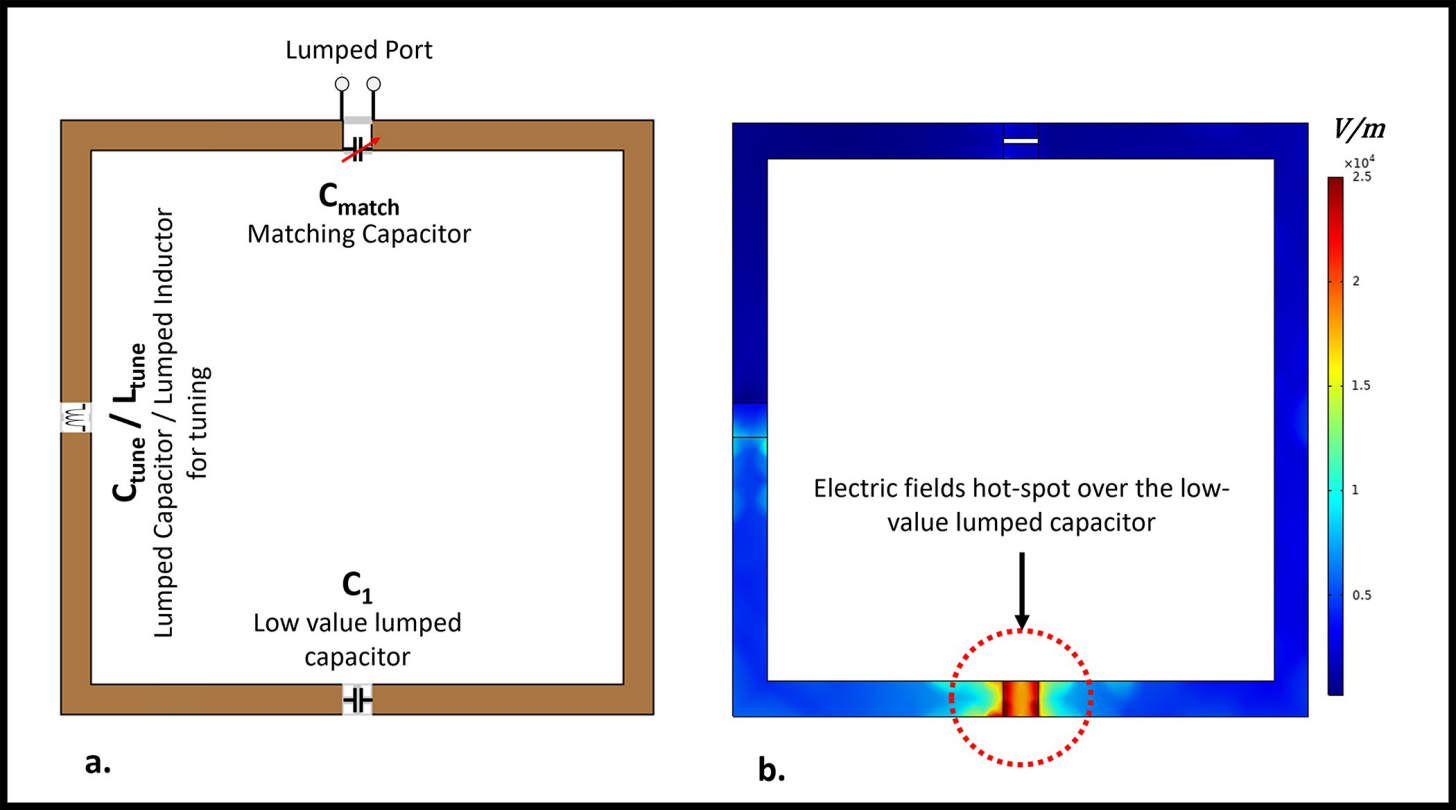
(a) Circuit schematic of a high-impedance coil (b) Surface electric field distribution over the single high-impedance coil element. Higher electric fields are observed over the low-value lumped capacitor placed opposite the port.

**Fig 2:**
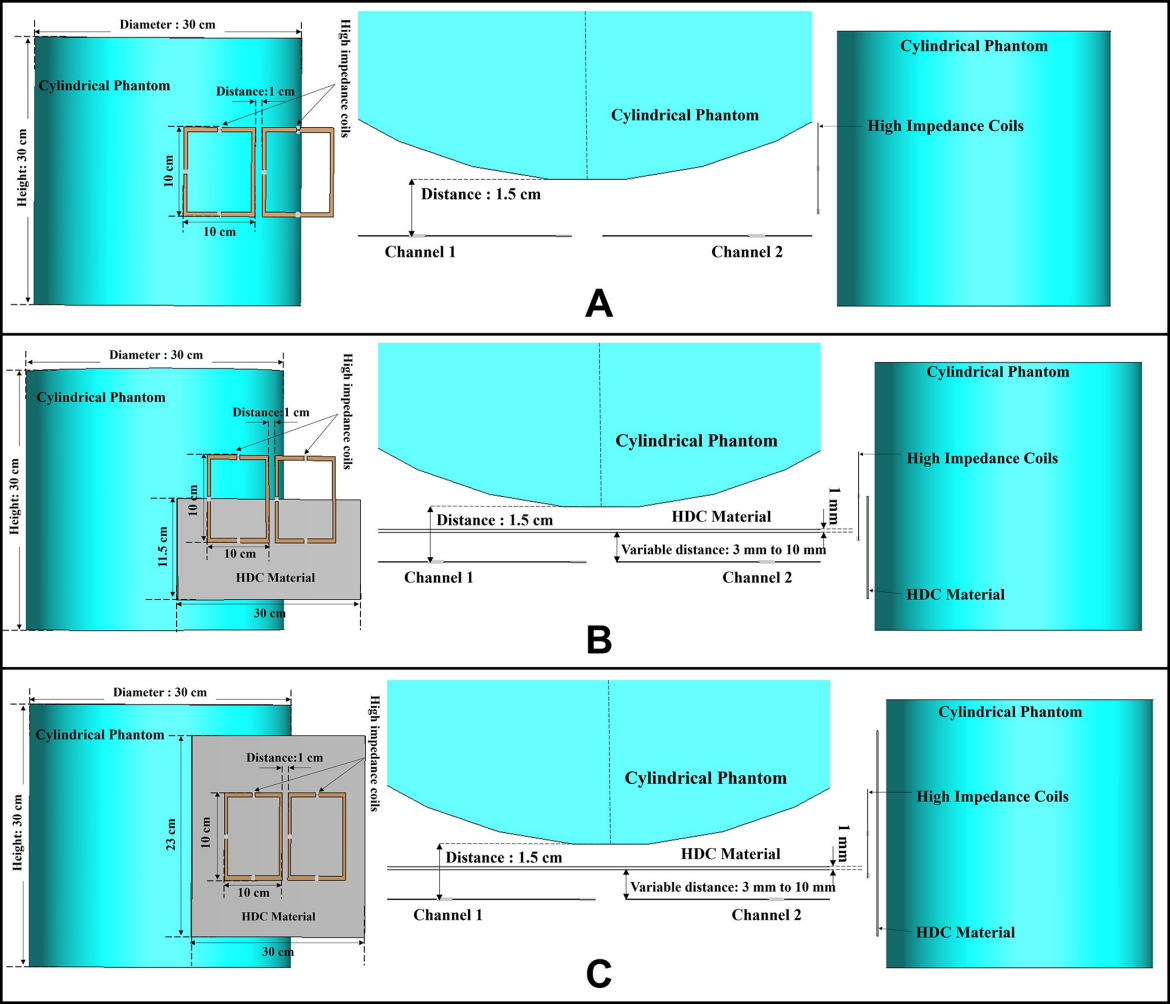
(a) Simulation setup for high-impedance coil without HDC material: 3D view with resonators near phantom, top view of components, side view. (b) Simulation setup for coils with 30 × 11.5 cm^2^ HDC sheet: 3D view with resonators and HDC sheet, top view of components, side view. (c) Simulation setup for coils with 30 × 23 cm^2^ HDC sheet: 3D view with resonators and HDC sheet, top view of components, side view.

Fig 1A depicts the circuit diagram of the high-impedance coil and the positioning of each lumped element on the coil. The low capacitance required for decoupling results in an increased E-field being generated across it. The surface electric field distribution over the high-impedance coil element is depicted in Fig 1B. Increased electric fields are observed close to the low-value lumped capacitor, as depicted in the picture. To address this issue, we tested numerous scenarios involving the use of high-dielectric-constant materials. The complete simulation setup used to evaluate the high-impedance coils without the high dielectric constant material can be seen in Fig 2A.

### B. 30 × 11.5 cm^2^ high dielectric constant material sheet partially covering the high impedance coils

Minor changes were made to the previous design by introducing a 30 × 11.5 cm^2^ high dielectric constant (HDC) material sheet with a thickness of 1 mm between the cylindrical phantom and the high-impedance coils. Fig 2B depicts the complete simulation setup for the case. Phantom’s material properties remained consistent with the previous design. The distance between the phantom and the high-impedance coils was kept at 1.5 cm, as in the previous case. The HDC material was positioned between these two components, with its distance from the high-impedance coils varying from 3 mm to 8 mm. The relative permittivity of the HDC material was modified, and several values ranging from 50 to 200 were utilized to evaluate the material’s impacts on inter-element isolation and other factors. When the HDC material is placed in front of the high-impedance coil, it introduces a dielectric load to the resonator, causing the tuning frequency to shift by a small margin. As a result, adjusting the impedances responsible for matching the tuning frequency for each situation suitably is critical.

The high-impedance coils were easily fine-tuned at 300 MHz by changing the Xarm impedances, but it was also clear that the low-value Cmode capacitor needed to be changed to find its appropriate combination with the Xarm inductors to enable the high-impedance coil’s self-decoupling property. The shortest distance between the HDC material and the resonator tested was 3 mm, and the others were 5 mm, 8 mm, and 10 mm. The distances were varied to compare the reductions in electric fields based on the distance from the HDC material. The varying relative permittivity values tested for each distance were due to challenges posed by the dielectric loading on the resonator, particularly in cases where the HDC material was in close proximity. For distances of 3mm and 5mm between the coils and the HDC material, relative permittivity values exceeding 100 would hinder frequency tuning at 300MHz and impedance matching to 50 ohms. Therefore, we specifically evaluated relative permittivity values of 50 and 100 for these distances. Similarly, for the 8mm distance between the coils, relative permittivity values exceeding 150 would present challenges in frequency tuning and impedance matching. Hence, we selected relative permittivity values of 50, 100, and 150 for evaluation at this distance. Finally, for the 10mm distance, challenges arose with relative permittivity values exceeding 200, impacting frequency tuning and impedance matching. Therefore, we assessed relative permittivity values of 50, 100, 150, and 200 for this distance. This approach ensured that we could perform fair and accurate comparisons while addressing the practical constraints posed by the experimental setup. The experiment attempted to reduce the higher electric field generation by adding a thin layer of HDC material over the coil at a specific distance while keeping the geometry and placement of each lumped component on the coil unchanged. The values of the lumped components used to tune the high-impedance resonators tuned at 300 MHz and matched at 50 ohms are listed in Table 1. Schematic circuit co-simulation was used to identify the combination of Cmode and Xarm impedances at which the self-decoupling behavior of the resonators was observed.

**Table 1:** Lumped element values of the high-impedance coil as a function of the 30 × 11.5 cm^2^ HDC material distance and permittivity.

Distance	relative permittivity (*ε*_*r*_)	Cmode (pF)	Xarm (nH)	Cmatch (pF)
3 mm	50	0.2355 pF	15.5 nH	16.3 pF
	100	0.1 pF	22 nH	15.4 pF
5 mm	50	0.24 pF	32 nH	15.4 pF
	100	0.11 pF	45 nH	15 pF
8 mm	50	0.2 pF	44 nH	15.7 pF
	100	0.15 pF	54.6 nH	15.1 pF
	150	0.09 pF	62.7 nH	14.8 pF
10 mm	50	0.265 pF	42 nH	15.7 pF
	100	0.195 pF	52 nH	15.1 pF
	150	0.125 pF	63 nH	15 pF
	200	0.09 pF	68 nH	14.7 pF

### C. 30 × 23 cm^2^ high dielectric constant material sheet completely covering the high-impedance coils

The dimensions of the high dielectric constant material sheet used in the previous design were increased to 30 × 23 cm^2^ while keeping the thickness the same at 1 mm. The rest of the simulation setup, including the cylindrical phantom and the high-impedance coils, remains consistent with previous cases. Similar to the prior case, the following distances between the HDC material and the high-impedance resonators were tested: 3 mm, 5 mm, 8 mm, and 10 mm, and the relative permittivity values at which the resonators tune at 300 MHz and preserve the decoupling performance were tested depending on the proximity of the HDC material to the resonators. Fig 2C shows the simulation setup used for the evaluation of the 30 × 23 cm^2^ HDC material covering the high-impedance coils completely while placed at a certain distance from the resonators. Following the same approach as the previous instance, each case evaluation required tuning the high-impedance resonators at 300 MHz and impedance matching at 50 ohms. Table 2 shows the values that were employed in combination to achieve frequency tuning and self-decoupling behavior.

**Table 2:** Lumped element values of the high-impedance coil as a function of the 30 × 23 cm^2^ HDC material distance and permittivity.

Distance	Relative permittivity (*ε*_*r*_)	Cmode (pF)	Xarm (nH)	Cmatch (pF)
3 mm	50	0.265 pF	2 nH	17.4 pF
	100	0.15 pF	4 nH	16.1 pF
5 mm	50	0.245 pF	25 nH	17 pF
	100	0.145 pF	33 nH	15.5 pF
8 mm	50	0.265 pF	35 nH	16.7 pF
	100	0.18 pF	45 nH	15.4 pF
	150	0.1 pF	57 nH	14.7 pF
10 mm	50	0.265 pF	40.8 nH	15.5 pF
	100	0.195 pF	50.5 nH	15.2 pF
	150	0.15 pF	54.5 nH	14.5 pF
	200	0.11 pF	59.28 nH	14.1 pF

### D. Construction and bench test measurements

Using a 3D printer, a 3D model of the 10 ×10 cm^2^ high-impedance coil was created. To replicate the coil design, the copper tape was employed as the conductor and adhered to the printed PLA (ε_r_: 2.8) former. In addition, the Cmode was a low-value trimmer capacitor (Johanson Giga-trim JK-272 0.4–2.5pF) that was mounted opposite the coaxial line feed. For tuning the coil at 300 MHz, a 10-pF variable capacitor was employed as an Xarm impedance. A shunt capacitor was also added to the feed line to match the coil’s impedance at 50 ohms, much like in the numerical simulation setup. We created a 3D-printed tray with the dimensions 28 × 22 cm^2^ and 28 × 11 cm^2^ to cover the high-impedance coil models to recreate the simulation scenario. Each of the printed trays was then covered with a 1 mm coating of gelatin and distilled water solution to make an HDC material with approximate relative permittivity (ε_r_) of 78.

**Fig 3:**
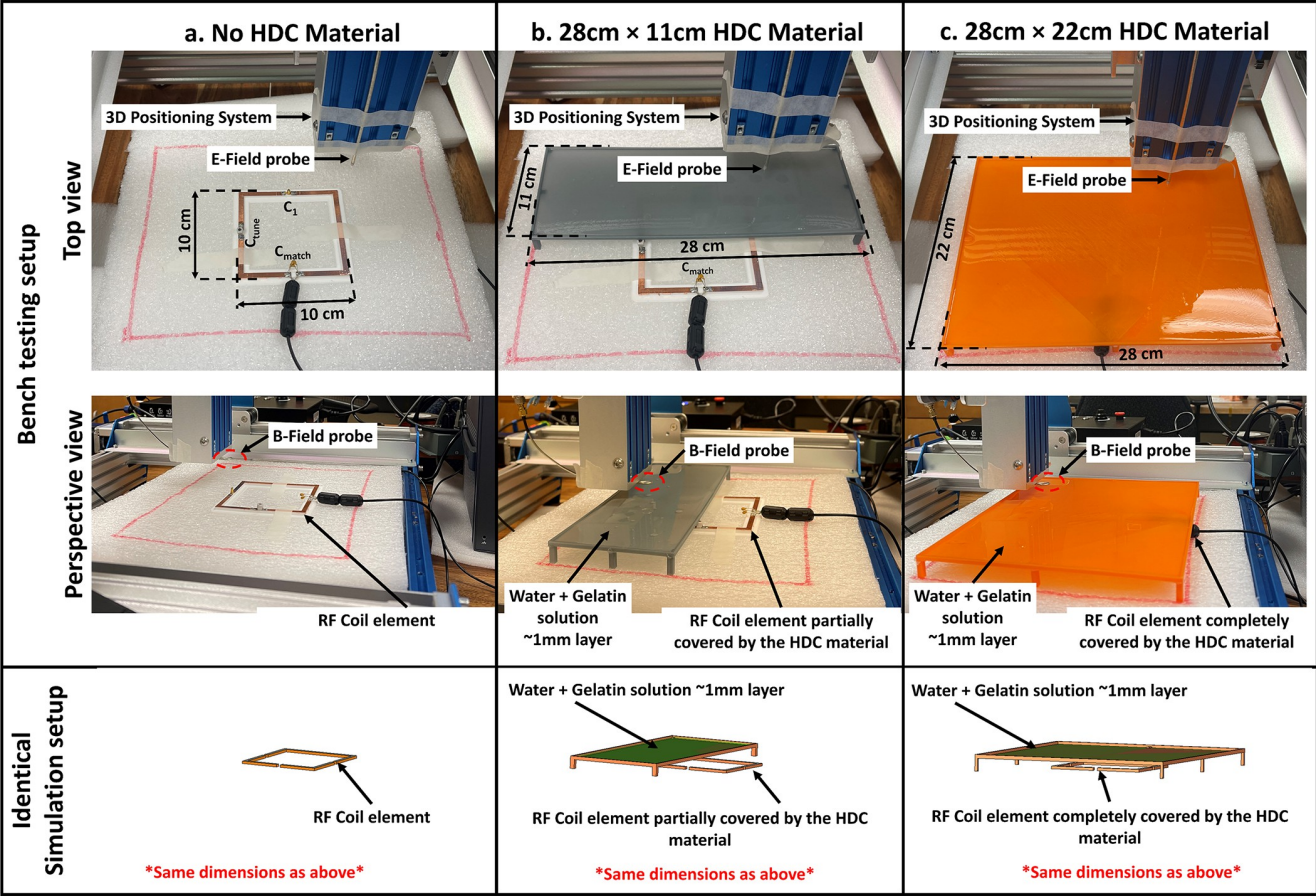
Bench test measurement setup and the identical simulation setup used for verification. (a) The high-impedance coil without any high dielectric constant material placed on top is kept on the low loss platform on the 3D positioning system and field measuring probe. (b)The high-impedance coil and the 3D printed tray containing the water + gelatin solution mimic one of the high relative permittivity values partially covering the high-impedance coil below. The partially covering 3D printed tray had the dimensions of 11×28 cm^2^ and the distance between the high dielectric constant material from the high-impedance coil was approximately 10cm. (c)The high impedance coil and 22×28 cm^2^ 3D printed tray with water + gelatin solution added on top. The distance between the high dielectric constant material and the high-impedance coil was approximately 10cm.

It is worth noting that no phantoms were used in the bench testing. The fields were meticulously mapped on a transverse plane located 15 mm from the high-impedance coil. To validate the experimental results, an identical simulation with the inclusion of the 3D model used for printing the tray which closely resembled the bench testing setup was performed. The simulated results were then compared to the measured results, yielding a thorough examination of the agreement between theoretical predictions and actual experimental outcomes. The bench testing arrangement and the appropriate simulation setup for every case investigated are shown in Fig 3. The H-field and E-field probes were connected to a 3D positioning system that was constructed using a high-precision CNC router (Genmitsu PROVerXL 4030) to map the corresponding fields above the high-impedance coil. The field mappings were evaluated based on the transmission coefficient S-parameters acquired through the probes at different points in a specified plane above the coil. The raw data transmission and the reflection coefficient values were obtained using the vector network analyzer (Keysight, E5061B, Santa Clara, CA, USA) and the data were further processed using MATLAB to acquire the field maps.

### E. Multi-channel array configuration

**Fig 4:**
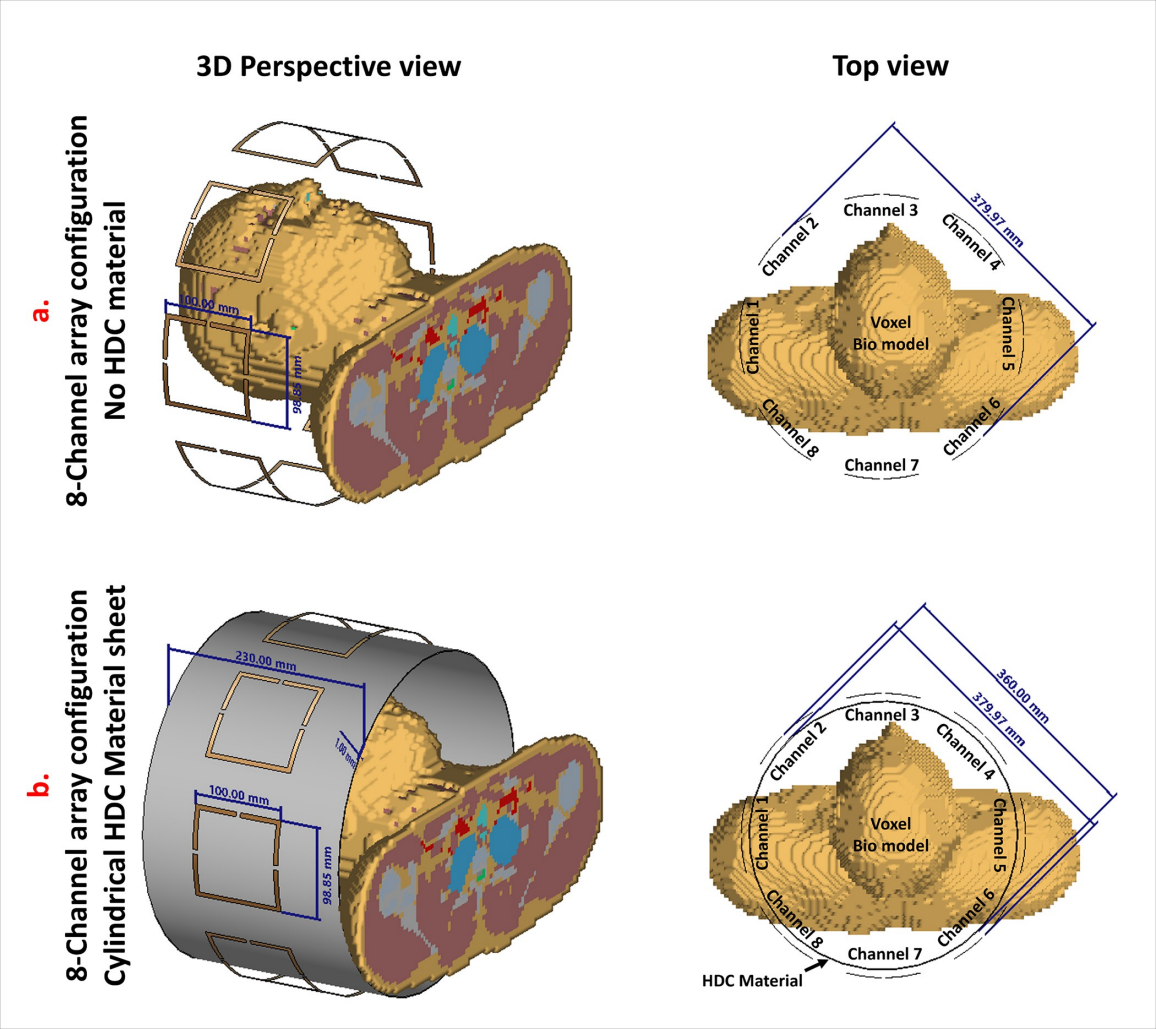
3D perspective and 2D top view of the voxel simulation setup used for evaluating the 8-channel array configuration, with and without High-Dielectric Constant (HDC) material. The human head voxel bio-model serves as the phantom: (a) Array without HDC material and (b) Array with HDC material.

Numerical simulations were conducted to examine the distribution of B1 and SAR in the Duke Human Bio Model using frequency domain and transient solvers [49–53]. The simulations involved 8-channel array configurations of high-impedance coils, both with and without the HDC material. The array was designed for 7T human brain imaging. The high-impedance coils were bent in a cylindrical shape and arranged in a circular configuration with a diameter of 380mm around the voxel model of the human head. The array configuration was assessed using a cylindrical HDC material sheet that had a thickness of 1mm, a diameter of 360mm, and a length of 230mm. The distance between each coil element and the HDC material was 10mm. The HDC material sheet had a relative permittivity of 50 and 200. The two array configurations assessed using a transient solver are depicted in Fig 4. In the array design without the HDC material, the following lumped components were employed to match and tune each coil element: Cmode = 0.21pF, Xarm = 76nH, and Cmatch = 13pF. The lumped components used to match and tune each coil element in the array design with the HDC material of relative permittivity 50 were as follows: Cmode = 0.22pF, Xarm = 52nH, and Cmatch = 12.25pF while the lumped components used to match and tune each coil element in the array design with the HDC material of relative permittivity 200 were as follows: Cmode = 0.162pF, Xarm = 52nH, and Cmatch = 12.25pF.

Furthermore, the same array configurations were evaluated using a frequency domain solver for a cylindrical homogeneous phantom to determine the SAR distribution, B1 field distribution, electric fields on the phantom surface, and inter-element isolation. The supporting documentation contains the evaluation.

## Results

### A. Inter-element isolation

**Fig 5:**
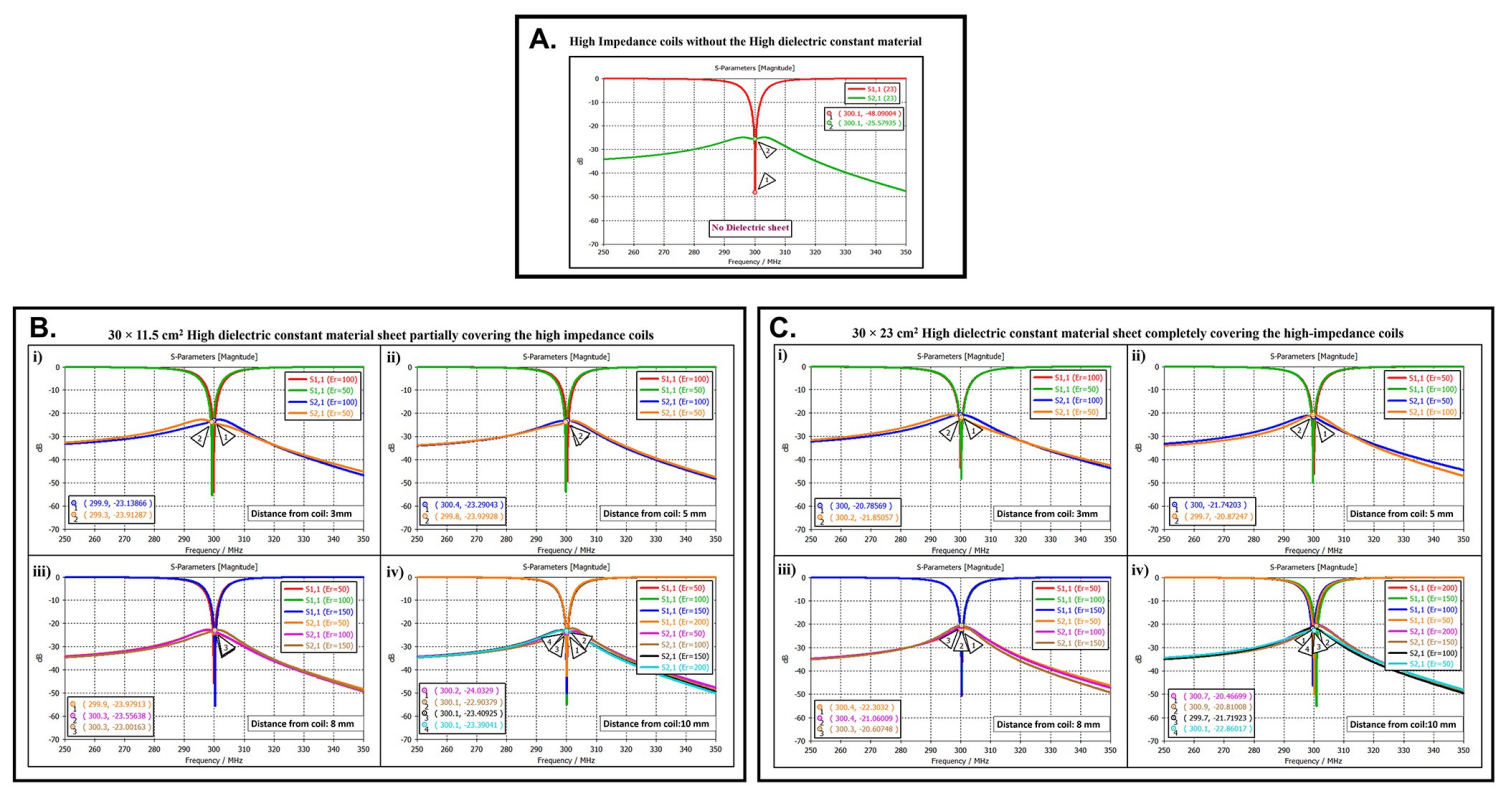
(a) Scattering parameters demonstrate reflection and transmission coefficients of high-impedance coils without HDC material. (b) Scattering parameters for experimental cases with 30 × 11.5 cm^2^ HDC material partially covering high-impedance coils: (i) HDC material placed 3mm away, (ii) HDC material placed 5mm away, (iii) HDC material placed 8mm away, (iv) HDC material placed 10mm away. (c) Scattering parameters for experimental cases with 30 × 23 cm^2^ HDC material completely covering high-impedance coils: (i) HDC material placed 3mm away, (ii) HDC material placed 5mm away, (iii) HDC material placed 8mm away, (iv) HDC material placed 10mm away.

The scattering parameters were used to calculate the isolation values between the high-impedance coils. Fig 2 depicts the simulation setup for each scenario under consideration. Following the previous procedure, the high-impedance coils were separated by 1 cm. The only modifications in the design were related to the relative permittivity of the HDC material used and the distance from the coil while maintaining a constant distance between the phantom and high-impedance coils in each scenario. With no HDC material present, the inter-element isolation value of the high-impedance coils was -25.57 dB. The S-parameter linear plot for high-impedance coils without HDC material is shown in Fig 5A. We used this value as the gold standard to compare the inter-element isolation values of different circumstances involving the application of HDC material to determine the effect the HDC material had on the isolation performance. The inter-element isolation values for high-impedance coils with HDC material above them were also evaluated. Fig 5B depicts the scattering parameters for the high-impedance coils, with a 30 × 11.5 cm^2^ HDC material sheet partially covering them.

The simulation setup shown in Fig 2B was used for the evaluations. The HDC material was placed between the high-impedance coils at distances ranging from 3 mm to 10 mm, with 3 mm being the closest. The HDC material had relative permittivity values of 50 and 100 at a distance of 3 mm, with inter-element isolation values of -23.9 dB and -23.1 dB, respectively. The HDC material with relative permittivity values of 50 and 100 was used for a 5 mm distance to achieve inter-element isolation values of -23.9 dB and -23.2 dB, respectively. Furthermore, the relative permittivity values for the HDC material were 50, 100, and 150 for an 8 mm distance, and the inter-element isolation values were -23.9 dB, -23.5 dB, and -23 dB, respectively. For the longest distance tested, 10 mm, the HDC material had relative permittivity values of 50, 100, 150, and 200, and the inter-element isolation values were -24 dB, -22.9 dB, -23.4 dB, and -23.3 dB, respectively. The inter-element isolation for each variable case tested for the 30 × 11.5 cm^2^ HDC material sheet partially covering the high-impedance coils was at least -20 dB, preserving the high-impedance coils’ inter-element isolation performance.

The scattering parameters for the other 30 × 23 cm^2^ HDC material sheet that completely covered the high-impedance coils are shown in Fig 5C. As with the previous HDC material sheet, the distance between the high-impedance coils on the given HDC material sheet was increased from 3 mm to 10 mm. For each distance tested, different relative permittivity values were used to validate the inter-element isolation performance of the high-impedance coils. Relative permittivity values of 50 and 100 were used for a distance of 3mm between the HDC material and the high-impedance coils, with inter-element isolation values of -21.8 dB and -20.7 dB, respectively. The HDC material with relative permittivity of 50 and 100 was also used for the 5mm distance, with corresponding inter-element isolation values of -21.7 dB and -20.8 dB. At a distance of 8 mm from the high-impedance coils, the HDC material was evaluated using relative permittivity values of 50, 100, and 150. In that order, the inter-element isolation values were -22.3 dB, -21 dB, and -20 dB. The relative permittivity values used were 50, 100, 150, and 200, with corresponding inter-element isolation values of -22.8 dB, -21.7 dB, -20.8 dB, and -20.4 dB for the 10 mm distance. The use of the HIC material produced inter-element isolation values of -20 dB or better between the high-impedance coils used in both evaluated cases, preserving the coils’ isolation performance.

### B. Electric fields

To assess the effect of HDC material on electric field values and distribution over the phantom, electric fields were computed using 3D electromagnetic simulations. A cylindrical phantom was included in the simulation setup to facilitate this, and different human tissue material properties were assigned to the phantom to investigate the electric field behavior of the high-impedance coils with the HDC material for various human tissue properties. The cylindrical phantom’s tissue properties included values for the human brain, kidney, breast fat, and tendon/ligament. The mentioned human tissue samples were chosen to facilitate the evaluation of various relative permittivity and electrical conductivity values. The material properties of the Brain phantom were as follows: relative permittivity Ɛ_r_: 50, electrical conductivity 0.6 S/m, and density 1000 Kg/m^3^. The Kidney phantom’s material properties were as follows: relative permittivity Ɛ_r_: 70.5, electrical conductivity 1.02 S/m, and density 1066 Kg/m^3^. The material properties of the Breast fat phantom were as follows: relative permittivity Ɛ_r_: 5.54, electrical conductivity: 0.0327 S/m, and density: 911 Kg/m^3^.Finally, the Tendon/Ligament phantom material properties were as follows: relative permittivity Ɛ_r_: 48, electrical conductivity 0.537 S/m, and density 1142 Kg/m^3^.

**Fig 6:**
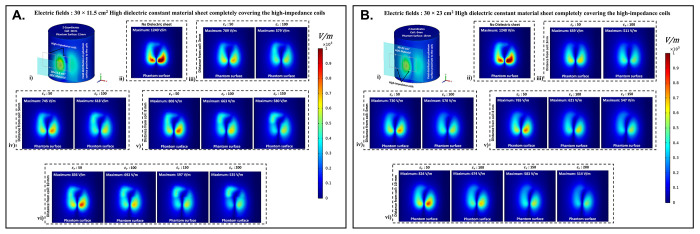
(a) Electric field distribution on cylindrical phantom with 30×11.5 cm^2^ HDC material: (i) 3D setup with coils and HDC material overlay; (ii) HDC material-free coil; (iii)-(vi) E-field distribution for HDC material 3mm, 5mm, 8mm, and 10mm from coils. (b) Electric field distribution on cylindrical phantom with 30×23 cm^2^ HDC material: (i) 3D setup with coils and HDC material overlay; (ii) HDC material-free coil; (iii)-(vi) E-field distribution for HDC material 3mm, 5mm, 8mm, and 10mm from coils.

The electric field distribution on the surface of the cylindrical phantom assigned with human brain tissue properties for the high-impedance coils covered with HDC materials of 30 × 11.5 cm^2^ dimension is shown in Fig 6A. When placed without any HDC material between them and the phantom, the high-impedance coils produced a peak electric field value of 1240 V/m on the phantom surface. The peak electric field values for the HDC materials kept 3 mm away from the coils were 709 V/m and 579 V/m for the relative permittivity of 50 and 100, respectively. Furthermore, for a 5mm distance between the coil and the HDC material, the peak electric field values of 745 V/m and 618 V/m for the relative permittivity of 50 and 100, respectively, were obtained. When the HDC material was 8 mm away from the high-impedance coils, the peak electric field values were 808 V/m, 663 V/m, and 580 V/m for the relative permittivity of 50, 100, and 150, respectively. Finally, for the largest distance evaluated (10 mm), peak electric fields of 836 V/m, 692 V/m, 597 V/m, and 535 V/m were observed for relative permittivity of 50,100,150, and 200, respectively. As a result, when HDC material with a relative permittivity of 200 was used 10 mm away from the high-impedance coils, the lowest peak electric field values (535 V/m) were observed.

Fig 6B depicts the electric field distribution on the surface of the cylindrical phantom for high-impedance coils made of HDC materials with dimensions of 30 × 23 cm^2^. The human brain material properties were assigned to the cylindrical phantom for electric field distribution evaluation, as in the previous case. The peak electric field value for high-impedance coils without HDC remains constant at 1240 V/m. Peak electric field values of 659 V/m for the relative permittivity of 50 and 511 V/m for the relative permittivity of 100 were observed when the HDC material was 3 mm away from the high-impedance coils. Peak electric field values of 730 V/m for the relative permittivity of 50 and 570 V/m for the relative permittivity of 100 were observed for the HDC material placed 5 mm away from the high-impedance coils. Furthermore, the peak electric field value was 765 V/m for the relative permittivity of 50, 621 V/m for the relative permittivity of 100, and 547 V/m for the relative permittivity of 150 for an 8 mm distance between the material and the coil. Finally, the peak electric field value was 824 V/m for the relative permittivity of 50, 674 V/m for the relative permittivity of 100, 583 V/m for the relative permittivity of 150, and 514 V/m for the relative permittivity of 200 for the farthest distance evaluated, which was 10 mm.

**Fig 7:**
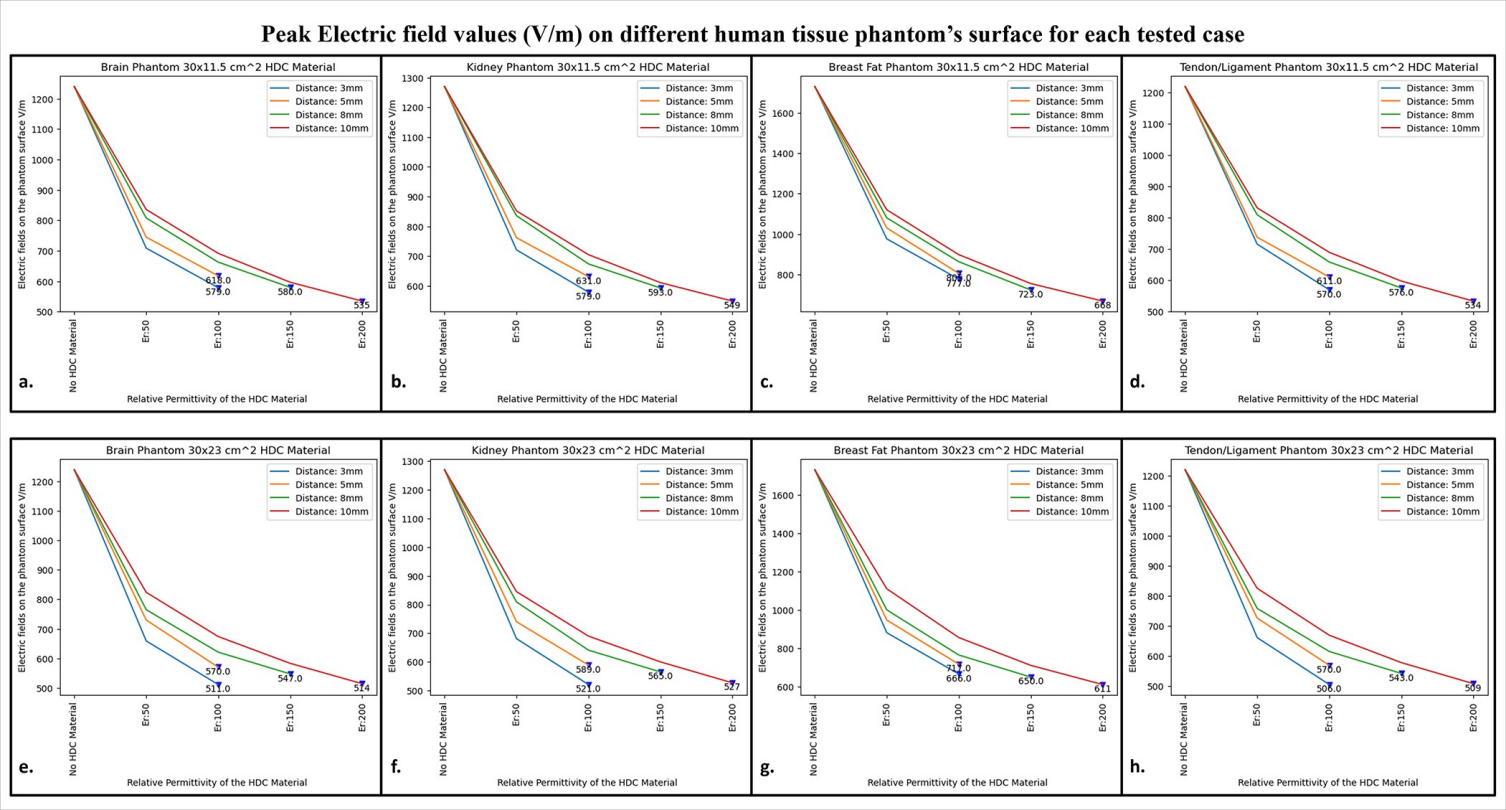
The 1D profiles of the peak electric field strengths evaluated for various cases involving different topologies of the HDC material, its distance from the high-impedance coils, its relative permittivity, and various human tissue properties assigned to the cylindrical phantom. (a) 30× 11.5 cm^2^ HDC material: Brain Phantom (b) 30× 11.5 cm^2^ HDC material: Kidney Phantom (c) 30× 11.5 cm^2^ HDC material: Breast Fat Phantom (d) 30× 11.5 cm^2^ HDC material: Tendon/ligament Phantom (e) 30× 23 cm^2^ HDC material: Brain Phantom (f) 30× 23 cm^2^ HDC material: Kidney Phantom (g) 30× 23 cm^2^ HDC material: Breast Fat Phantom (h) 30× 23 cm^2^ HDC material: Tendon/ligament Phantom.

As a result, when the HDC material with a relative permittivity of 100 was kept 3 mm away from the coil, the lowest peak electric field value of 511 V/m was observed. Other human tissue properties, such as kidney, breast fat, and tendon/ligament, were assigned to the cylindrical phantom, and similar cases were evaluated to obtain peak electric field values on the phantom surface. Fig 7 depicts one-dimensional profiles for the peak electric field trend for each human tissue parameter evaluated. For each tissue property, the peak electric field values on the phantom surface decrease in the same pattern. When a partially covering HDC material with dimensions of 30 × 11.5 cm^2^ was used and kept at a distance of 10 mm from the high impedance coils, the maximum reduction in peak electric field values was observed for all of the evaluated tissue properties. Furthermore, when another topology of HDC material with dimensions of 30 × 23 cm^2^ was used, the brain, kidney, and tendon/ligament phantoms had a significant decrease in peak electric field strength when HDC material with a relative permittivity of 100 was used, and kept 3 mm away from the coils. In addition, for the Breast fat phantom, the HDC material with a relative permittivity of 200 and a distance of 10 mm from the coils showed a significant reduction in peak electric fields on the phantom surface.

The peak electric field values were reduced by 56.85% when a partially covering high dielectric constant material measuring 30×11.5 cm^2^ was used. Additionally, when high dielectric constant materials measuring 30×23 cm^2^ were used to completely cover the RF coils, the peak electric field values were reduced by 58.54%. These reductions were observed in a phantom with properties assigned to mimic human brain tissue. The 30×11.5 cm^2^ high dielectric constant material sheet resulted in a reduction of the peak electric field values for the kidney, breast fat, and tendon/ligament by 57.16%, 61.38%, and 56.22%, respectively. Furthermore, kidney, breast fat, and tendon/ligament tissue properties showed a 58.97%, 64.68%, and 58.27% reduction in peak electric field values when the RF coils were fully covered by a 30×23 cm^2^ high dielectric constant material sheet.

### C. Specific absorption rate

**Fig 8:**
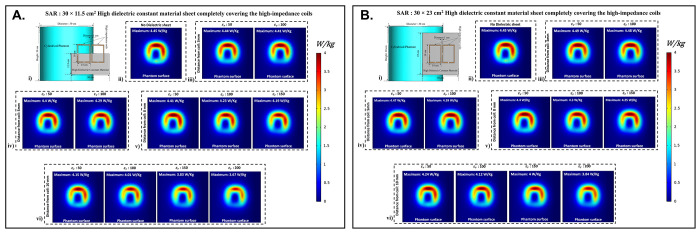
(a) Specific Absorption Rate (SAR) distribution on cylindrical phantom with 30×11.5 cm^2^ HDC material: (i) Simulation setup for SAR evaluation; (ii) HDC material-free coil SAR distribution; (iii)-(vi) SAR distribution for HDC material 3mm, 5mm, 8mm, and 10mm from coils. (b) Specific Absorption Rate (SAR) distribution on cylindrical phantom with 30×23 cm^2^ HDC material: (i) Simulation setup for SAR evaluation; (ii) HDC material-free coil SAR distribution; (iii)-(vi) SAR distribution for HDC material 3mm, 5mm, 8mm, and 10mm from coils.

Using electromagnetic simulations, the peak specific absorption rate values (W/Kg) in the cylindrical phantom assigned with human tissue properties were calculated. The cylindrical phantom used in the simulations was assigned similar human tissue properties such as the human brain, kidney, breast fat, and tendon/ligament, and the SAR distribution and peak SAR values were recorded for all of the stated tissue properties. The SAR distribution on the cylindrical phantom assigned the human brain tissue properties, as well as the peak SAR values for the respective field distribution for the high impedance coils covered with 30 × 11.5 cm^2^ HDC material, are shown in Fig 8A. The peak SAR value of the high-impedance coils without HDC materials was 4.45 W/kg. The evaluated cases for the HDC material with dimensions of 30 × 11.5 cm^2^ and a distance from the high impedance coils ranging from 3mm to 10mm, on the other hand, had the following peak SAR values: Peak SAR values of 4.44 and 4.41 W/Kg were observed for the material with relative permittivity of 50 and 100, respectively, for an evaluated distance of 3mm between the HDC material and the high impedance coils. Similarly, for the material with relative permittivity of 50 and 100, peak SAR values of 4.4 and 4.29 W/Kg were observed for the 5 mm evaluated distance between the material and the coils, respectively. Furthermore, for a distance of 8mm, the peak SAR values for the material with relative permittivity of 50,100, and 150 were 4.41,4.23, and 4.19 W/Kg, respectively. Finally, for the material with relative permittivity of 50,100,150, and 200, peak SAR values of 4.15,4.01,3.83,3.67 W/Kg were observed for the evaluated distance of 10mm. Similar cases were evaluated for the HDC material with different dimensions of 30 × 23 cm^2^ following the same pattern.

Fig 8B depicts the SAR field distribution, as well as the peak SAR values recorded for each evaluated case using HDC material with dimensions of 30 × 23 cm^2^. Peak SAR values of 4.49 and 4.48 W/Kg were observed for the material with relative permittivity of 50 and 100 at a 3mm distance between the material and the high-impedance coils, respectively. Similarly, for a 5mm distance, the peak SAR values were 4.47 and 4.39 W/Kg for materials with relative permittivity of 50 and 100, respectively. Furthermore, for the 8mm distance, peak SAR values of 4.4,4.3,4.25 W/Kg were obtained for materials with relative permittivity values of 50, 100, and 150, respectively. Finally, for the material with relative permittivity of 50,100,150, and 200, peak SAR values of 4.24,4.12,4, and 3.84 W/Kg were recorded for the maximum distance evaluated of 10mm. Peak SAR values for other human tissue properties such as kidney, breast fat, and tendon/ligament were also evaluated for cylindrical phantoms.

**Fig 9:**
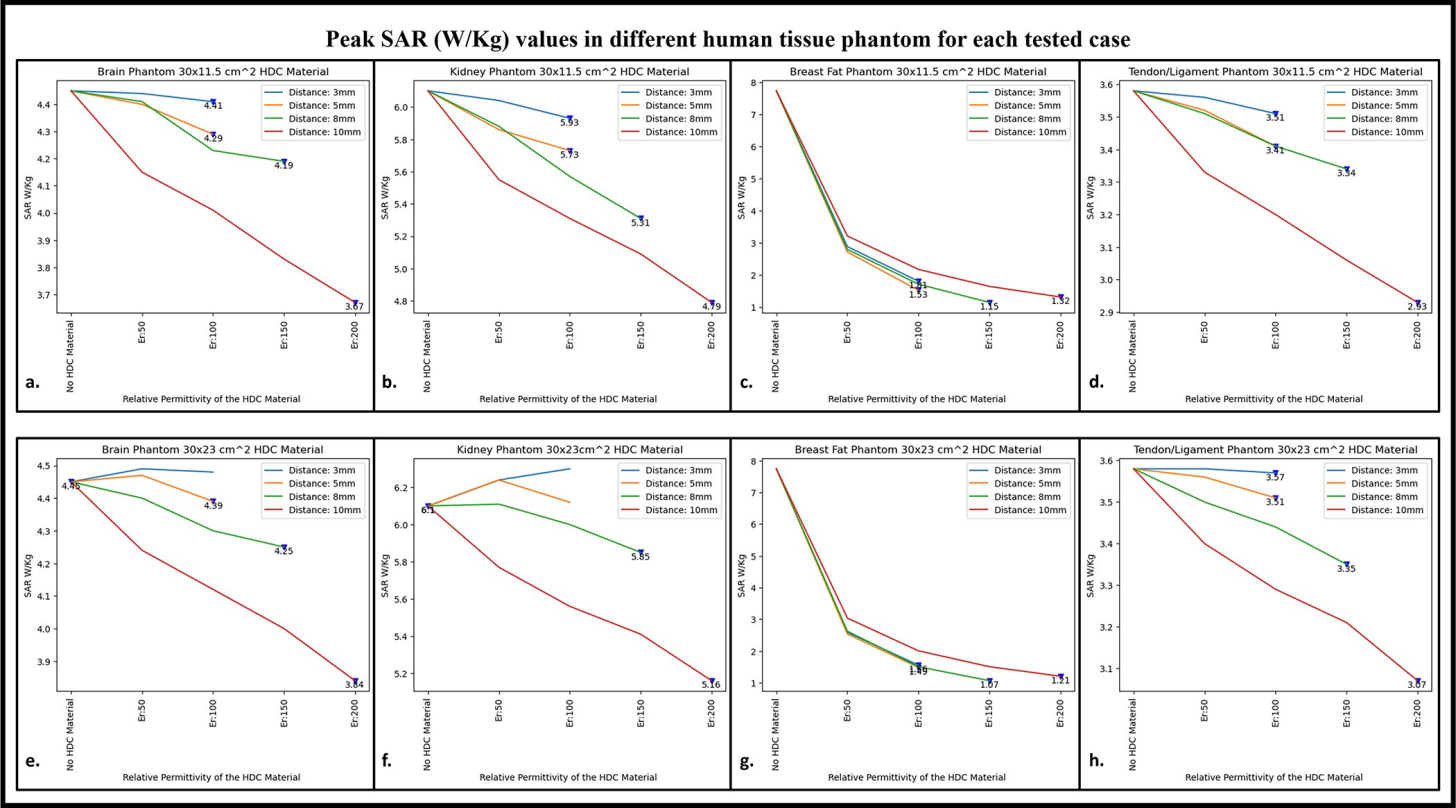
The 1D profiles of the peak SAR values evaluated for various cases involving different topologies of the HDC material, its distance from the high-impedance coils, its relative permittivity, and various human tissue properties assigned to the cylindrical phantom. (a) 30× 11.5 cm^2^ HDC material: Brain Phantom (b) 30× 11.5 cm^2^ HDC material: Kidney Phantom (c) 30× 11.5 cm^2^ HDC material: Breast Fat Phantom (d) 30× 11.5 cm^2^ HDC material: Tendon/ligament Phantom (e) 30× 23 cm^2^ HDC material: Brain Phantom (f) 30× 23 cm^2^ HDC material: Kidney Phantom (g) 30× 23 cm^2^ HDC material: Breast Fat Phantom (h) 30× 23 cm^2^ HDC material: Tendon/ligament Phantom.

Fig 9 depicts the 1D profiles of peak SAR values for all of the cases studied, which involved HDC material sheets with varying relative permittivity values and varying distances between them and the high-impedance coils. The selected human tissue properties include a wide range of relative permittivity and electric conductivity values found inside the human body and will provide additional insights into SAR value behavior based on relative permittivity and electric conductivity values. When placed without any HDC material in between, the high impedance coils deposited peak SAR values of 4.45 W/Kg, 6.1 W/Kg, 7.74 W/Kg, and 3.58 W/Kg in the brain, kidney, breast fat, and tendon/ligament phantoms, respectively. After inserting the HDC material with dimensions of 30 × 11.5 cm^2^ between the phantom and the coils, the peak SAR value in the brain phantom was reduced to 3.67 W/Kg, 4.79 W/Kg in the kidney, 1.32 W/Kg in the Breast fat phantom, and 2.93 W/Kg in the Tendon/ligament phantom. In a similar pattern, for the HDC material with dimensions of 30 × 23 cm^2^, the peak SAR value for the brain phantom was reduced to 3.84 W/Kg, the peak SAR value for the kidney phantom was reduced to 5.16 W/Kg, the peak SAR value for the breast fat was reduced to 1.21 W/Kg, and the peak SAR value for the tendon/ligament was reduced to 3.07 W/Kg. The utilization of high dielectric constant material, with dimensions of 30×11.5 cm^2^, resulted in a reduction of peak specific absorption rate (SAR) values by 17.52%. Similarly, when the high dielectric constant materials, with dimensions of 30×23 cm^2^, completely covered the RF coils, a reduction of peak SAR values by 13.70% was observed. These evaluations were conducted on a phantom model that simulated human brain tissue properties. The high dielectric constant material sheet with dimensions of 30×11.5 cm^2^ resulted in peak SAR value reductions of 21.47%, 85.14%, and 18.15% in kidney, breast fat, and tendon/ligament tissue properties, respectively. Furthermore, the peak SAR values for kidney, breast fat, and tendon/ligament tissue properties were reduced by 15.40%, 86.17%, and 14.24%, respectively, by the 30×23 cm^2^ high dielectric constant material sheet that covered the entire RF coils.

### D. B1 field distribution

**Fig 10:**
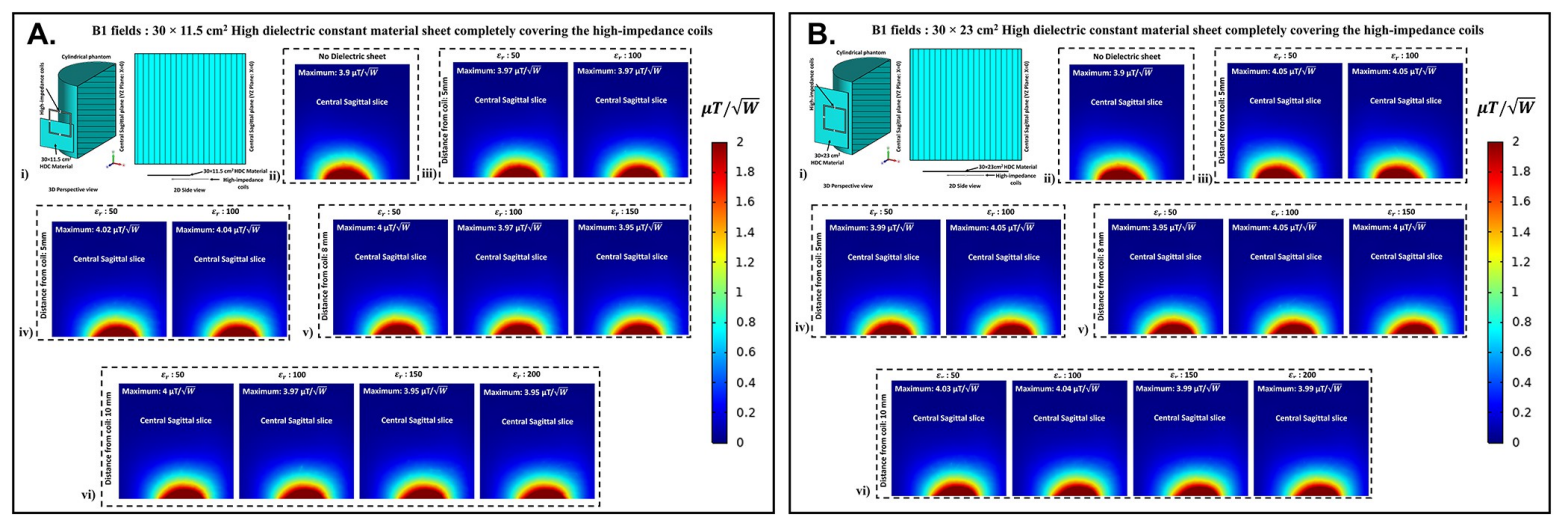
(a) B1 field distribution on central sagittal cross-section of cylindrical phantom with 30×11.5 cm^2^ HDC material: (i) 3D and 2D perspectives of central sagittal cut plane, illustrating B1 field distribution within phantom; (ii) HDC material-free coil B1-field distribution; (iii)-(vi) B1-field distribution for HDC material 3mm, 5mm, 8mm, and 10mm from coils. (b) B1 field distribution on central sagittal cross-section of cylindrical phantom with 30×23 cm2 HDC material: (i) 3D and 2D perspectives of central sagittal cut plane, illustrating B1 field distribution within phantom; (ii) HDC material-free coil B1-field distribution; (iii)-(vi) B1-field distribution for HDC material 3mm, 5mm, 8mm, and 10mm from coils.

B1 field distribution in the central sagittal slice of the cylindrical phantom assigned with human brain tissue properties was computed to assess how the presence of HDC material sheet between the phantom and the high-impedance coils affects the B1 field efficiency of the high-impedance coils. Fig 10A displays the high-impedance coils’ B1 field distribution in the center sagittal slice of the cylindrical phantom, which is partially covered by a sheet of HDC material with measurements of 30 × 11.5 cm^2^. Each analyzed case’s peak B1 field values were also assessed in the central sagittal slice to track the pattern of the peak B1 field strength with its distribution. Without using any HDC material, the high-impedance coils’ initial B1 field distribution and peak B1 value were calculated. For a fair comparison, the computed B1 field strength values in μT were normalized by dividing by the square root of the accepted power for the high-impedance coils in each analyzed scenario. Peak B1 values of 3.9 $\mu T/\sqrt{W}$ were generated by the high-impedance coils devoid of HDC material.

It was intended to compare this beginning value to the peak B1 field values computed for each example using HDC material with a particular relative permittivity value to ascertain whether the HDC material had any adverse effects on the B1 field distribution of the high-impedance coils. Similar cases involving the use of HDC material with different relative permittivity values and distance from the high-impedance coils were evaluated for the B1 field distribution and peak B1 field strength in the cylindrical phantom’s central sagittal slice. For the HDC material kept 3mm away, two relative permittivity values of 50 and 100 were used, yielding peak B1 field strengths of 3.97 and 3.97 $\mu T/\sqrt{W}$, respectively. Similarly, for the HDC material 5mm away from the high-impedance coils, the same relative permittivity values (50 and 100) produced peak B1 field strengths of 4.02 and 4.04 $\mu T/\sqrt{W}$, respectively. Furthermore, for the HDC material placed 8mm away, relative permittivity values of 50,100, and 150 were used, yielding peak B1 field strengths of 4, 3.97, and 3.95 $\mu T/\sqrt{W}$, respectively. Finally, at a distance of 10mm between the HDC material and the high-impedance coils, relative permittivity values of 50,100,150, and 200 were used for the HDC material, with peak B1 field strengths of 4,3.97,3.95, and 3.95 $\mu T/\sqrt{W}$, respectively. The evaluated cases involving HDC material of dimensions 30×11.5 cm^2^ consistently produced peak B1 field strengths greater than the initial recorded value of 3.9 $\mu T/\sqrt{W}$ and preserved the high-impedance coil structure’s B1 field distribution. Similarly, for the experimental cases involving the use of HDC material with dimensions 30×23 cm^2^, the B1 field distribution in the sagittal slice of the cylindrical phantom assigned with human brain tissue properties was evaluated.

The B1 field distribution for all of the evaluated cases for the HDC material with the specified dimensions is shown in Fig 10B. For a distance of 3mm between the HDC material and the high-impedance coils, the HDC material was assigned two relative permittivity values of 50 and 100, and peak B1 field strengths of 4.05 and 4.05 $\mu T/\sqrt{W}$ were observed in the phantom’s central sagittal slice, respectively. Relative permittivity values of 50 and 100 were used for the evaluated distance of 5mm, and peak B1 field strengths of 3.99 and 4.05 $\mu T/\sqrt{W}$ were recorded, respectively. Furthermore, for the 8mm distance, the HDC material had relative permittivity values of 50,100, and 150, with peak B1 field strengths of 3.95, 4.05, and 4 $\mu T/\sqrt{W}$, respectively. Finally, for a 10mm distance, the HDC material had relative permittivity values of 50,100,150, and 200, with peak B1 field strengths of 4.03, 4.04, 3.99, and 3.99 $\mu T/\sqrt{W}$, respectively.

### E. Bench test results

**Fig 11:**
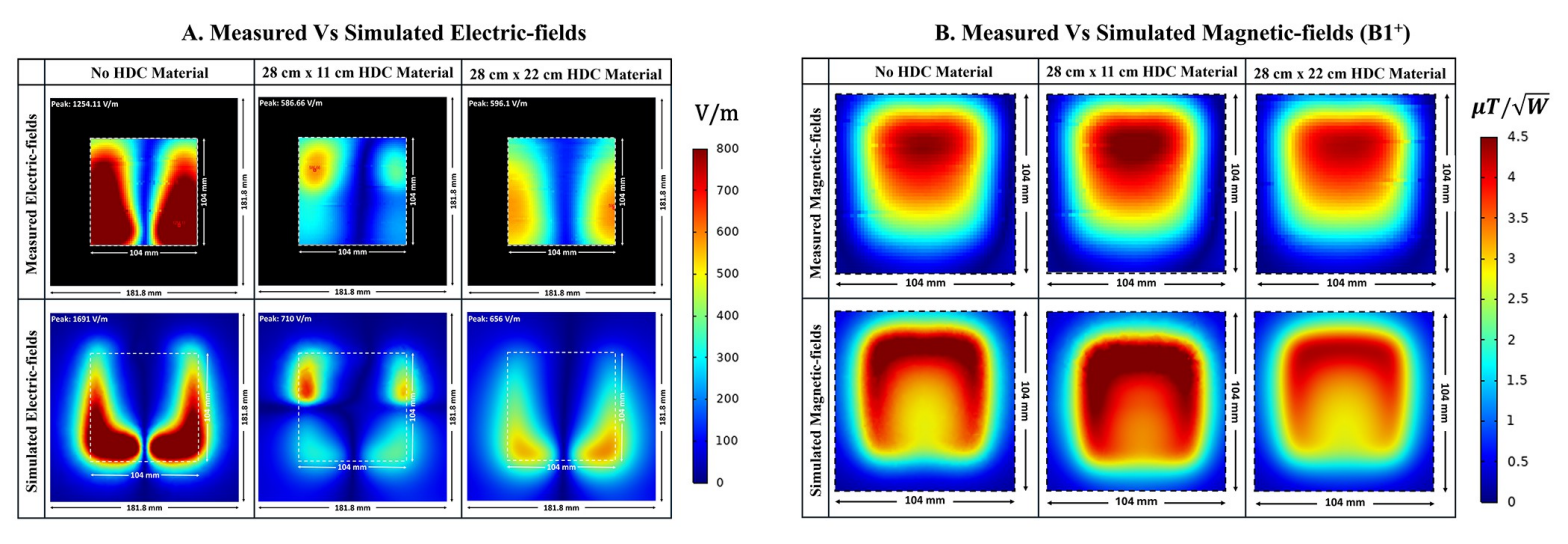
(a) Comparison of Electric Field Distributions: Measured and simulated electric fields within a 104mm x 104mm Field of View (FOV) for direct comparison. (b) Comparative Analysis of B1 Field Distributions: Measured and simulated B1 field distributions in an identical setup for each case.

The simulated results were validated using bench test results using the experimental setup shown in Fig 3. For all of the evaluated cases, the field distributions were plotted on an axial plane approximately 15 mm from the coil, and no phantom was used in simulations or bench tests. The measured and simulated electric field distributions for the three evaluated cases are shown in Fig 11A. High-impedance coils with no HDC material present, high-impedance coils with a 28×11 cm^2^ 3D printed tray with a 1mm thick HDC material layer added on top, and high-impedance coils with a 28×22 cm^2^ 3D printed tray with a 1mm thick HDC material layer added on top are among the cases. For each case, the field distributions were plotted in an axial plane 15mm away from the coil. In addition to the field distribution, the peak electric field strength was measured for each of the cases studied. In the bench testing setup, the first evaluated case involving high-impedance coils without any HDC material produced a peak electric field strength of 1254.11 V/m versus 1691 V/m in the simulation setup. Furthermore, in the bench testing setup, a high-impedance coil covered by the HDC material tray with dimensions of 28×11 cm^2^ produced a peak electric field strength of 586.66 V/m vs. 710 V/m in the simulation setup. Finally, in the bench testing setup, a high-impedance coil covered by the HDC material tray with dimensions of 28×22 cm^2^ produced a peak electric field strength of 596.1 V/m vs. 656 V/m in the simulation setup. Overall, the measured peak electric field value for the bench testing setup was reduced by 53.22% when HDC material added to the 3D printed tray with dimensions of 28×11 cm^2^ was placed above the high impedance coil, and it was reduced by 52.46% when HDC material added to the 3D printed tray with different dimensions of 28×22 cm^2^ was placed above the high impedance coil. In an identical simulation setup, the peak electric field strength was reduced by 58.01% with the application of HDC material with dimensions of 28×11 cm^2^ and by 61.20% with the addition of HDC material with dimensions of 28×22 cm^2^ above the high-impedance coil.

Magnetic field distribution in $\mu T/\sqrt{W}$ was compared between the experimental and simulation setups to further validate the concept. The B1 field distribution was evaluated in both the experimental bench testing setup and the simulation setup for three cases, including high-impedance coils with and without the HDC material placed on top of the coils (see Fig 11B). The measured and simulated results agreed well, with an improved overall B1 field strength for the case involving the application of HDC material with dimensions of 28×11 cm^2^ and a slightly reduced B1 field strength for the case involving the application of HDC material with dimensions of 28×22 cm^2^. The disparity between measured and simulated values can be attributed to a difference in the accuracy of the results between bench testing and simulation setup. The measured field distributions were reconstructed by measuring each field on an axial plane about 15 mm away from the coil. The measurement grid included a 51×51 matrix with a precision of 2mm, and the simulation setup included very fine mesh settings with a specified minimum element length of 0.1mm. The higher simulation accuracy resulted in overall higher field strength values. Another factor that contributes to the discrepancy is the relative permittivity of the HDC material used. The HDC material was created by combining gelatin and distilled water, and its relative permittivity was estimated to be around 78. However, the practical value of the HDC material could be higher or lower than the assumed value, resulting in higher peak electric field values in the simulated results.

### F. Multi-channel array configuration: Voxel B1 and SAR distribution

**Fig 12:**
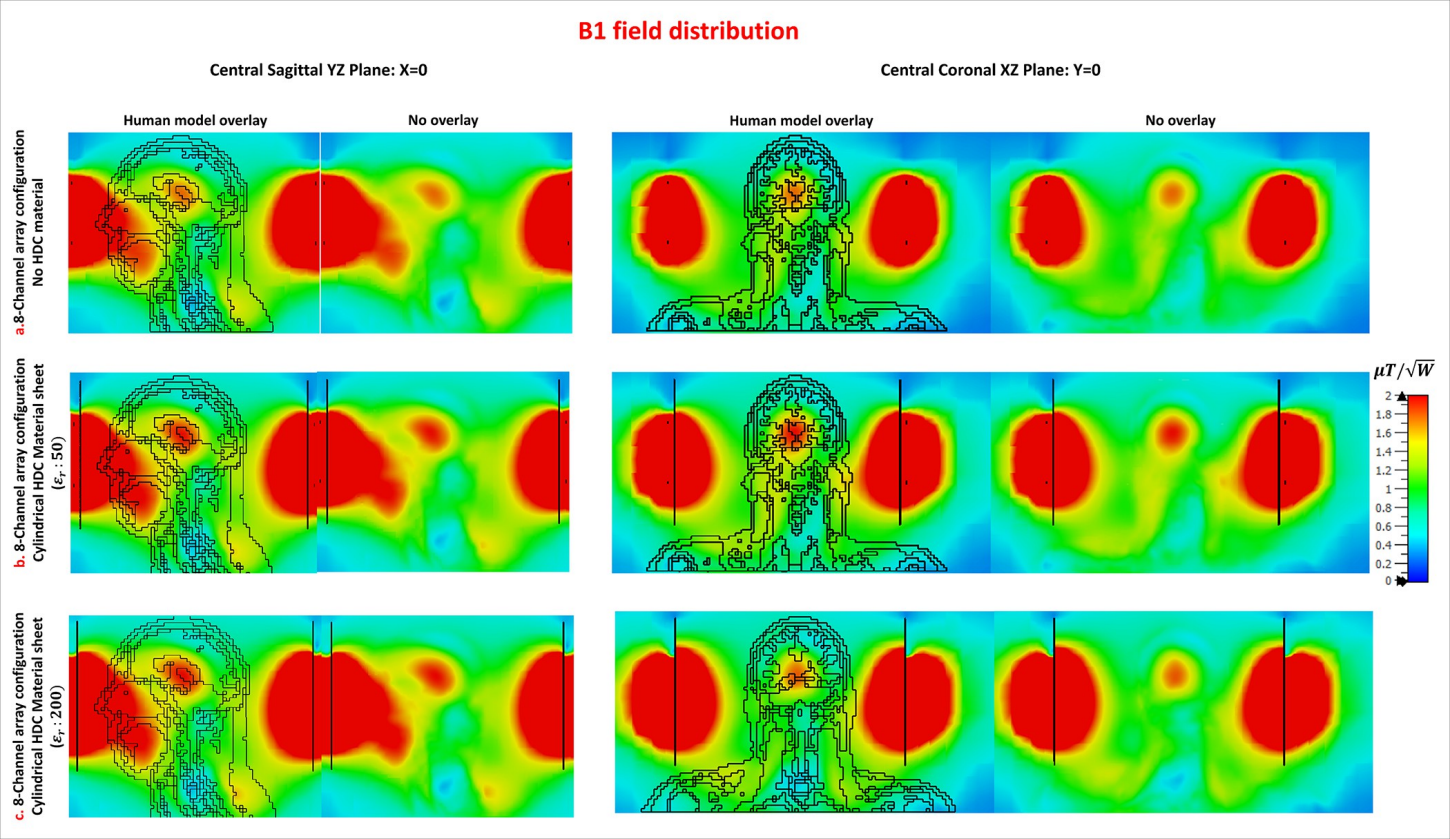
B1 field distribution evaluation in the voxel bio model for 8-channel array setups with and without the High Dielectric Constant (HDC) material sheet. The figure shows the B1 field distribution in coronal and sagittal planes at the center of the human model, highlighting the improved performance with HDC material.

In the voxel bio model, the B1 fields and SAR distribution of the array setups were evaluated with and without the High Dielectric Constant (HDC) material sheet. The B1 field distribution in several voxel model planes is shown in the accompanying Fig 12. The figure displays the B1 field distribution (represented in $\mu T/\sqrt{W}$) in the coronal and sagittal planes, with and without the human model overlay. In this set of simulations with the human model, the results show that the addition of the HDC material produced an improvement in the B1 fields. This implies a favorable effect on the system’s performance, highlighting the HDC material’s effectiveness in enhancing B1 field characteristics in the studied bio model.

**Fig 13:**
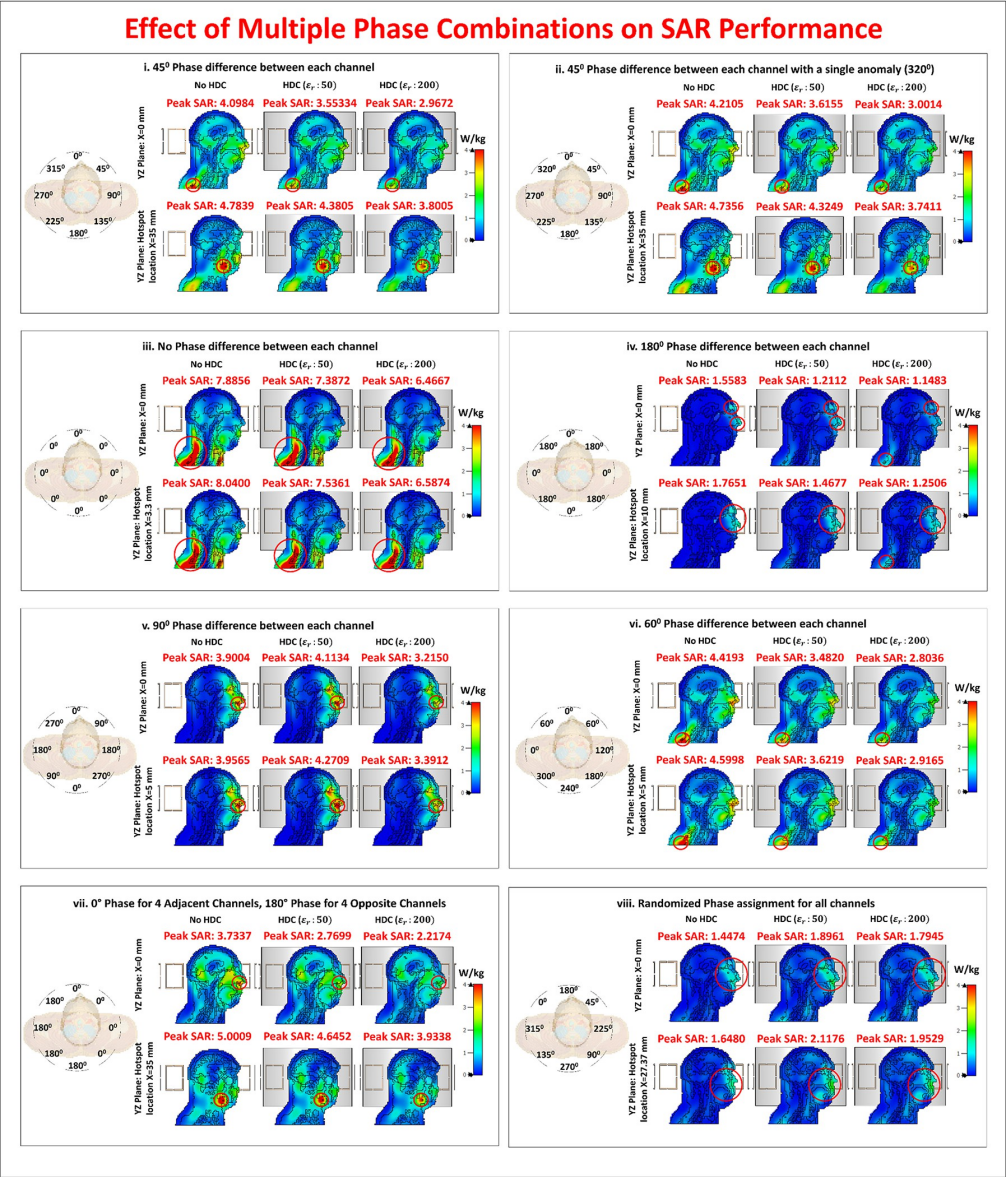
Effect of multiple phase combinations on SAR distribution and performance. SAR distributions in sagittal planes, with analysis. Peak SAR is reduced with HDC ε_r_: 50 and 200. Mean SAR values show reductions of 6.26% and 20.14%, respectively, when compared to no HDC material.

Furthermore, Fig 13 displays the distribution of the specific absorption rate (SAR) in the central sagittal plane and another sagittal plane location where maximum SAR hotspots were generated. Since SAR values depend on the phase of the individual channels of the array, different phase combinations were validated to compare the performance of the proposed method in each phase setting and demonstrate the SAR intensity and distribution are sensitive to the phase change of each channel of the coil array. The phase combinations used in the evaluations include a variety of configurations designed to assess different scenarios. The most common phase setting involves assigning phases according to the circular angle at which each channel is placed. For example, in an 8-channel setup, each channel would have a 45°-phase difference. Another combination maintains the 45°-phase difference but introduces a single anomaly: the phase assignment is 320° rather than the standard 315°. This anomaly simulates scenarios similar to B1 shimming, in which slight phase variations are used to achieve B1 homogeneity.

The third phase combination gives all channels the same phase, resembling a linear polarization scenario. The fourth combination shows alternating phase assignments of 0° and 180°. Subsequent combinations create 90° and 60°-phase differences between array channels, respectively. Another combination involves four channels with a 0°-phase opposite to four channels with an 180°-phase. The final combination consists of completely random phase assignments. It is important to note that, while some of these phase combinations may not be appropriate for practical MR image acquisition, they have been investigated to ensure safe performance in a variety of situations.

In the first phase combination (Fig 13i), an 8.43% peak SAR reduction (4.7839 W/kg vs. 4.3805 W/kg) was observed with HDC ε_r_: 50, and a 20.55% reduction (4.7839 W/kg vs. 3.8005 W/kg) with HDC ε_r_: 200. The second combination (Fig 13ii) showed an 8.67% peak SAR reduction (4.7356 W/kg vs. 4.3249 W/kg) with HDC ε_r_: 50, and a 21.00% reduction (4.7356 W/kg vs. 3.7411 W/kg) with HDC ε_r_: 200. In the third combination (Fig 13iii), HDC ε_r_: 50 resulted in a 6.26% peak SAR reduction (8.0400 W/kg vs. 7.5361 W/kg), while HDC ε_r_: 200 resulted in an 18.06% reduction (8.0400 W/kg vs. 6.5874 W/kg). The fourth combination (Fig 13iv) resulted in a 16.84% peak SAR reduction (1.7651 W/kg vs. 1.4677 W/kg) with HDC ε_r_: 50 and a 29.14% reduction (1.7651 W/kg vs. 1.2506 W/kg) with HDC ε_r_: 200.

The fifth combination (Fig 13v) showed a 7.94% peak SAR increase (3.9565 W/kg vs. 4.2709 W/kg) with HDC ε_r_: 50 and a 14.28% decrease (3.9565 W/kg vs. 3.3912 W/kg) with HDC ε_r_: 200. The sixth combination (Fig 13vi) showed a peak SAR reduction of 21.25% (4.5998 W/kg vs. 3.6219 W/kg) with HDC ε_r_: 50 and 36.59% (4.5998 W/kg vs. 2.9165 W/kg) with HDC ε_r_: 200. In the seventh combination (Fig 13vii), HDC ε_r_: 50 resulted in a 7.11% peak SAR reduction (5.0009 W/kg vs. 4.6452 W/kg), while HDC ε_r_: 200 resulted in a 21.33% reduction (5.0009 W/kg vs. 3.9338 W/kg). The final eighth combination (Fig 13viii) demonstrated a 28.49% peak SAR increase (1.6480 W/kg vs. 2.1176 W/kg) with HDC ε_r_: 50 and an 18.50% increase (1.6480 W/kg vs. 1.9529 W/kg) with HDC ε_r_: 200.

The mean SAR values for three cases (no HDC, HDC ε_r_:50, and HDC ε_r_:200) were calculated using peak SAR values from eight phase combinations. The mean SAR values were found to be 4.3162 W/kg for the case without HDC material, 4.0456 W/kg (6.26% reduction) for HDC ε_r_:50, and 3.4467 W/kg (20.14% reduction) for HDC ε_r_:200.

## Conclusions and discussions

In this work, we numerically and experimentally evaluated a novel method of incorporating high dielectric constant material to reduce electric fields and SAR values in high-impedance RF coils and arrays. Using numerical simulations, experimental cases introducing high dielectric constant material to high-impedance coils and strategic placement of the high dielectric constant material with appropriate relative permittivity to maximize E-field and SAR reduction were evaluated to demonstrate the proposed method’s success in lowering electric fields and SAR values over high-impedance coils. When compared to high dielectric constant material free high impedance coils, our proposed approach successfully reduced the peak electric field values by at least 50% and the SAR values by at least 13% in numerical simulations while preserving the initial B1 efficiency and decoupling performance of the high impedance coils. Furthermore, we ran numerical simulations on an 8-channel array configuration with and without the use of High Dielectric Constant (HDC) material with a relative permittivity of 50. The results showed a significant improvement in B1 performance and a reduction in peak Specific Absorption Rate (SAR) values, demonstrating the efficacy of our proposed method in an array configuration. Our findings indicate that further increases in the relative permittivity of HDC material have the potential to improve imaging performance. We are confident that the proposed method is scalable for higher channel numbers, and ongoing optimization of key parameters promises to yield even more robust results in future applications.

Our initial attempts with High-Dielectric Constant (HDC) material at the coil’s back did not produce adequate results in terms of decreasing electric fields and Specific Absorption Rate (SAR) values. The most successful arrangement used HDC material between the coil and the phantom. In response to concerns about losing 3–8 mm of inner radius, we feel that using HDC material as a spacer within the coil encasing is possible without compromising imaging performance. This method finds a balance between safety concerns and achieving optimal imaging findings. The electric field distribution case analyzed for HDC material at the coil’s back is included in the **S1 File.**

To reduce electric fields, we investigated scenarios with thicker HDC material sheets, but we encountered unique problems with this coil dimension. Our coil dimensions are suitable for a 300MHz LC loop (10x10 cm^2^). Any dimensions greater than this limit would make frequency matching more difficult. Reducing the distance between the coils and the HDC material sheet (either partially or completely covered) beyond the lower limit of 3mm would present additional impedance and frequency matching challenges for our experimental coil dimensions, as the dielectric load imposed by the HDC material would prevent the coils from reaching 300MHz and achieving the required inter-element isolation.

Another way to reduce the distance between the coils and the HDC material is to use smaller patches of HDC material with a higher relative permittivity and place them closer to the coils than the current 3mm limit. However, our results show that this method is less effective than using HDC materials with a lower permittivity but a larger coverage area over the coils. Our study identified several critical parameters for optimal electric field and SAR reduction: the relative permittivity of the HDC material, the size of the HDC material patches, the distance between the HDC material and the coils, and the thickness of the HDC materials. According to our findings and the specific dimensions of the LC loop-based high impedance coil used, keeping a distance of 3mm to 8mm between the HDC material and the coils, and completely or partially covering the coils with relatively thinner (1mm) HDC material, can result in a 50% reduction in electric fields over the phantom surface. However, we believe the space can be reduced for smaller coil sizes because the reduced inductance introduced by the coil itself would allow for the placement of HDC material closer to the coil while balancing the dielectric load presented by the HDC material and effectively matching the frequency and impedance.

Following the successful evaluation of the proposed method in numerical simulations, a prototype was tested on the bench to test one of the experimental setups, and the results demonstrated preservation of B1 efficiency and peak electric field value reduction of at least 50%, validating the simulation results. Our findings demonstrate the potential of high dielectric constant materials in ultra-high field MR imaging as a viable option for alleviating electromagnetic exposure concerns and enabling safer MR imaging at ultra-high fields. The use of high-dielectric constant materials for exposure reduction is not limited to high-impedance coils, but can also be used in conjunction with other RF coils. Further research into the use of high dielectric constant materials in MRI RF hardware will undoubtedly contribute to safer and more efficient electromagnetic systems.

## Supporting information

S1 File HDC material placement evaluation. (PDF)

S2 File Frequency domain simulations for multichannel array configurations. (PDF)
