# Supplementary material for: Electric field and SAR reduction in high-impedance RF arrays by using high permittivity materials for 7T MR imaging
Source: PLoS One. 2024 Jul 3;19(7):e0305464. doi: 10.1371/journal.pone.0305464 (PMC11221758; doi:10.1371/journal.pone.0305464)
Supplement: S1 File — (PDF) [file pone.0305464.s001.pdf]

HDC material placement evaluation

A numerical simulation instance in which the HDC material with a relative permittivity of 50 is placed behind the coil rather than between the coil and the phantom has been examined. The coil is 10mm away from the HDC material, and 15mm away from the phantom. The cylindrical phantom with material parameters of Ɛr: 50 and electrical conductivity 0.6 S/m was employed, with the phantom dimensions as follows: Dimensions: 30 cm in length and diameter. The HDC material utilized for the case evaluation measured 30×23 cm2 with a thickness of 1mm.

The results show that the peak electric field reduction was only 4.8% when the HDC material was kept at the back of the coil, compared to a 33% reduction when the HDC material was kept in front of the coil, between the coil and the phantom.

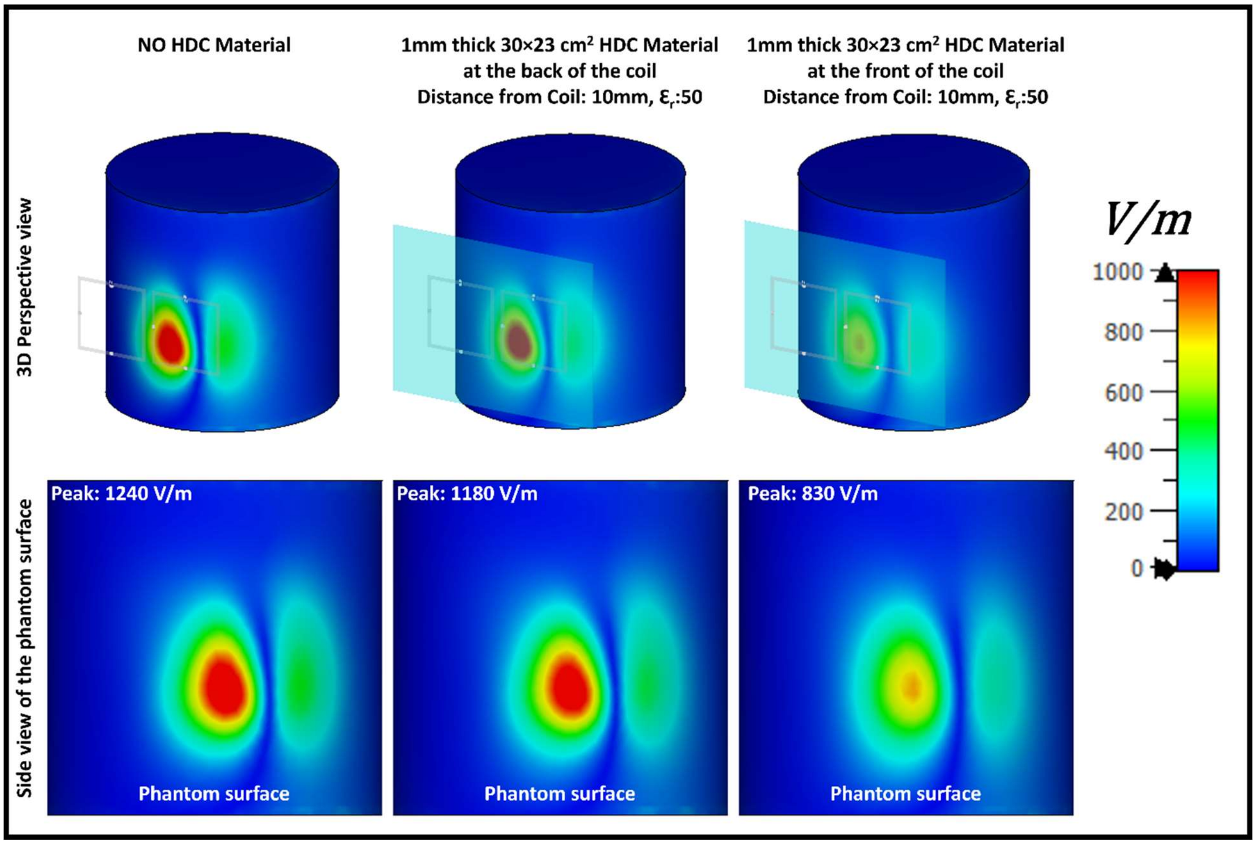

Fig 1. Electric field distribution on the surface of the cylindrical phantom for three evaluated cases: (1) Without High-Dielectric Constant (HDC) material, (2) With HDC material at the back of the coil, and (3) With HDC material in the front of the coil, positioned between the coil and the cylindrical phantom.
