# Supplementary material for: Electric field and SAR reduction in high-impedance RF arrays by using high permittivity materials for 7T MR imaging
Source: PLoS One. 2024 Jul 3;19(7):e0305464. doi: 10.1371/journal.pone.0305464 (PMC11221758; doi:10.1371/journal.pone.0305464)
Supplement: S2 File — (PDF) [file pone.0305464.s002.pdf]

Frequency domain simulations for multichannel array configurations

Phantom Material properties: ε_r: 50, electrical conductivity 0.6 S/m, and density 1000 Kg/m^3
Phantom Dimensions: Length: 30 cm, Diameter: 30 cm
HDC Material: ε_r: 50 and 200
HDC Material Dimensions: Diameter: 36 cm, Length: 23 cm

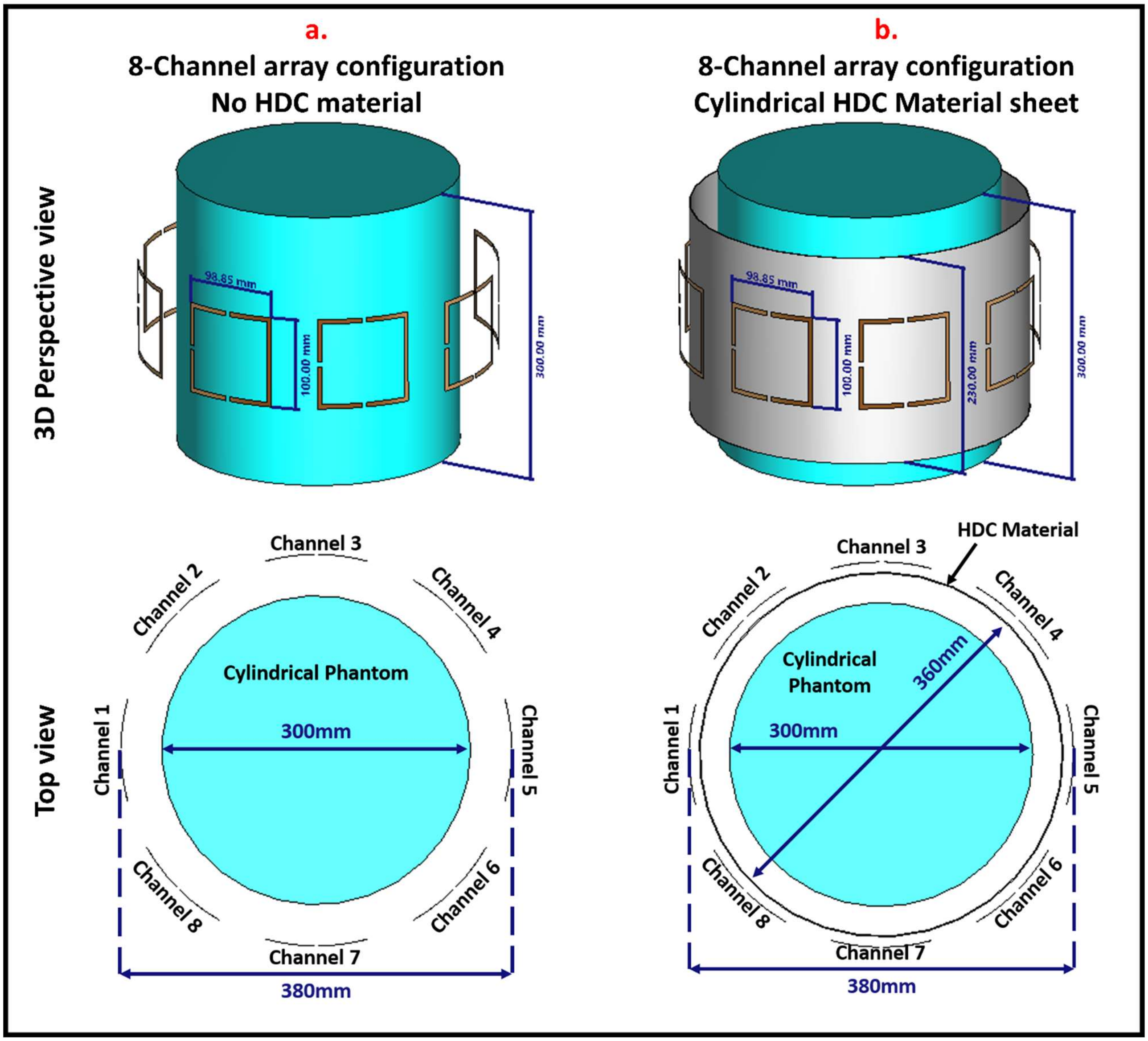

Fig 1. 3D perspective and 2D top view of two 8-channel array configurations: one without and one with a long cylindrical High-Dielectric Constant (HDC) material sheet. The figure schematic includes labeled coil element placement and dimensions for both configurations.

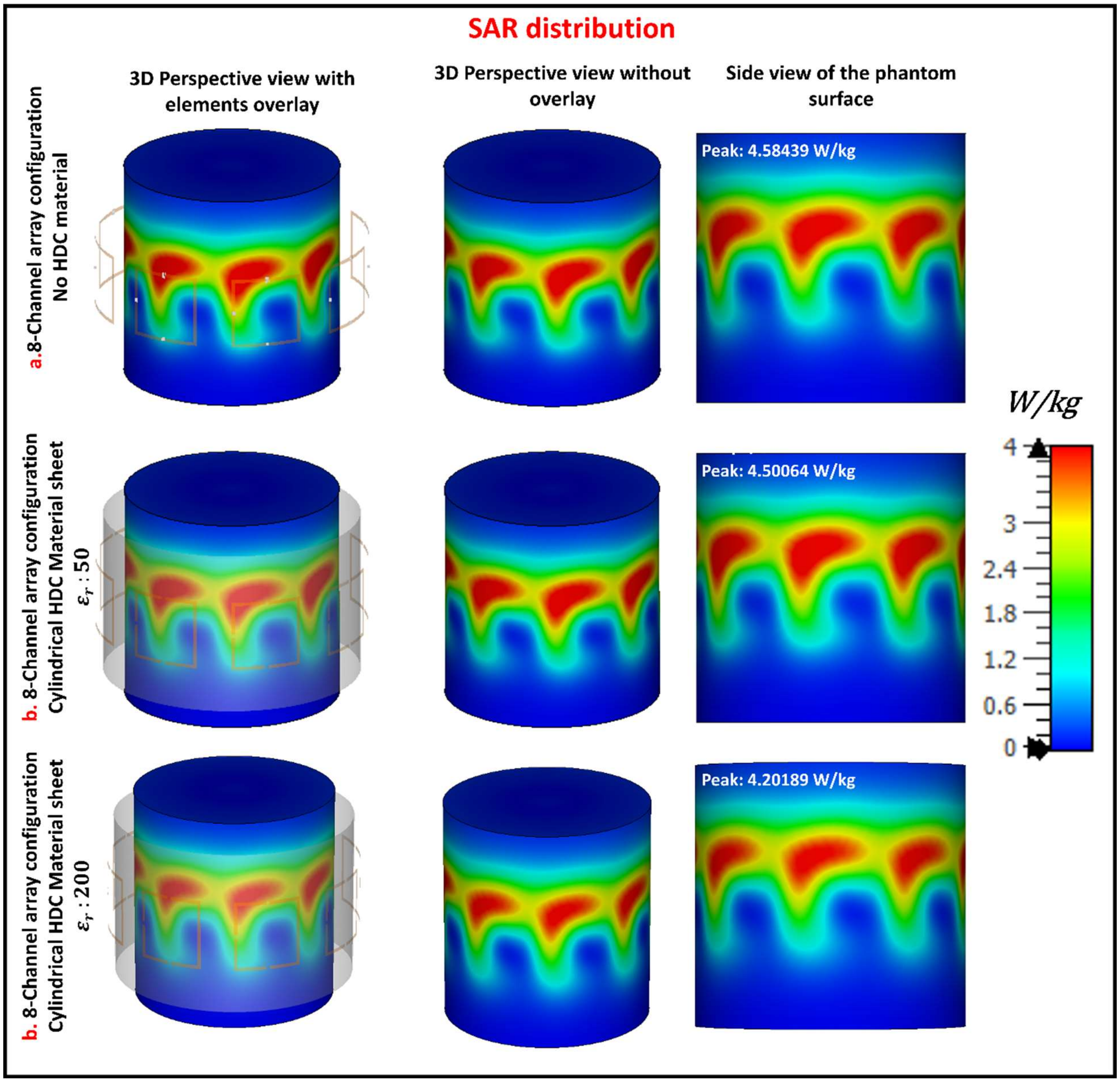

Fig 2. SAR distribution on the phantom surface for the array configurations depicted in three views: 3D perspective with an overlay of coils and High-Dielectric Constant (HDC) material, 3D perspective without overlay, and a side profile illustrating the SAR fields on the phantom surface.

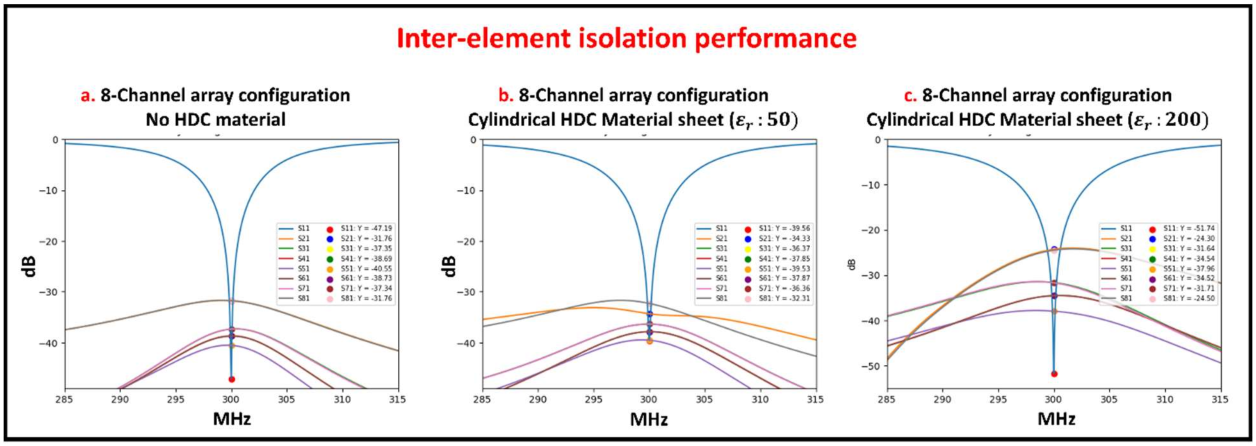

Fig 3. S-parameters plot illustrating reflection and transmission coefficient parameters for all coil elements employed in the array configurations, one without and others with the inclusion of the long cylindrical High-Dielectric Constant (HDC) material sheet (ε_r:50 and 200).

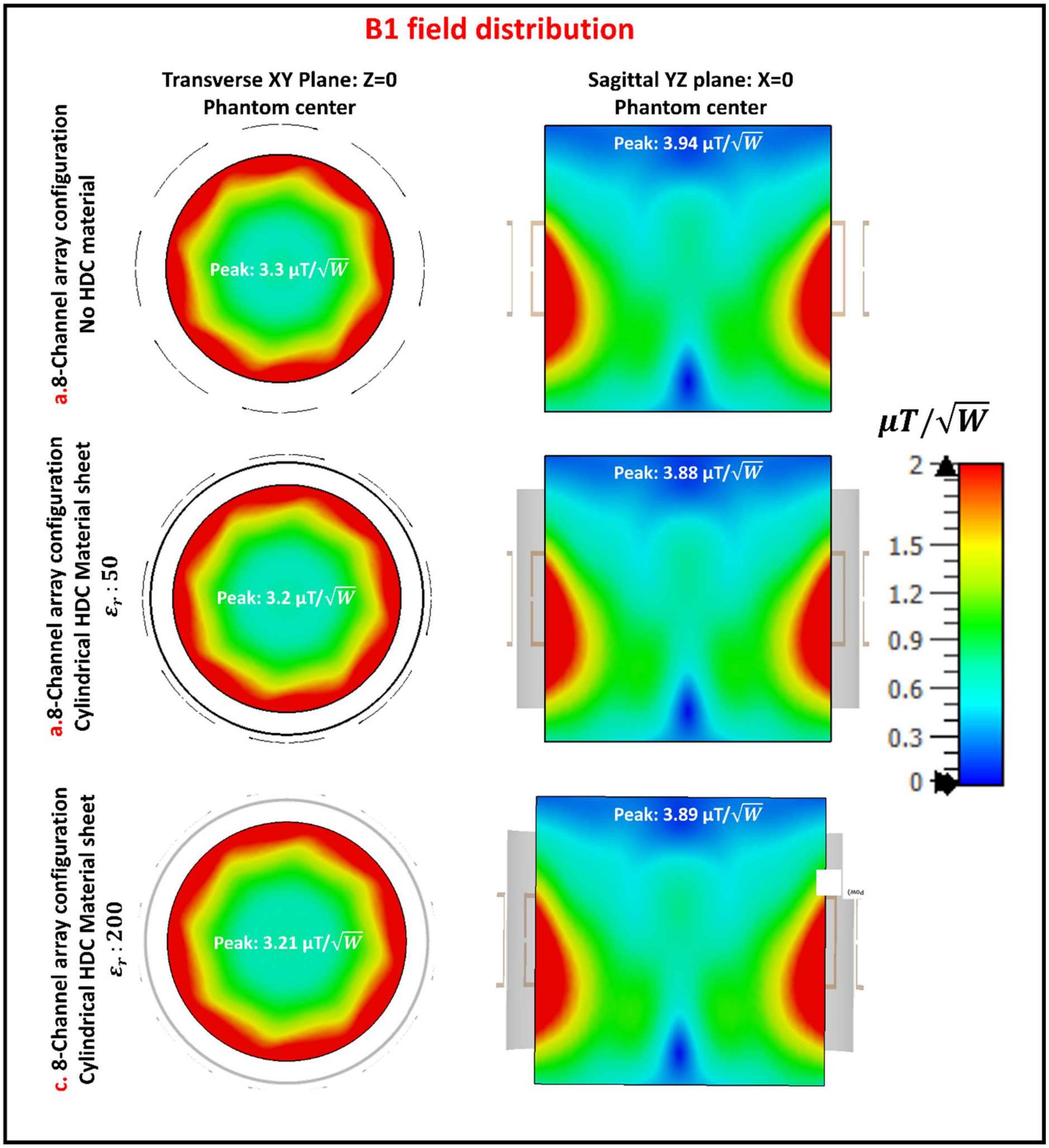

Fig 4. B1 field distribution for the array configurations in the central transverse plane of the phantom center and the central sagittal plane of the phantom center.

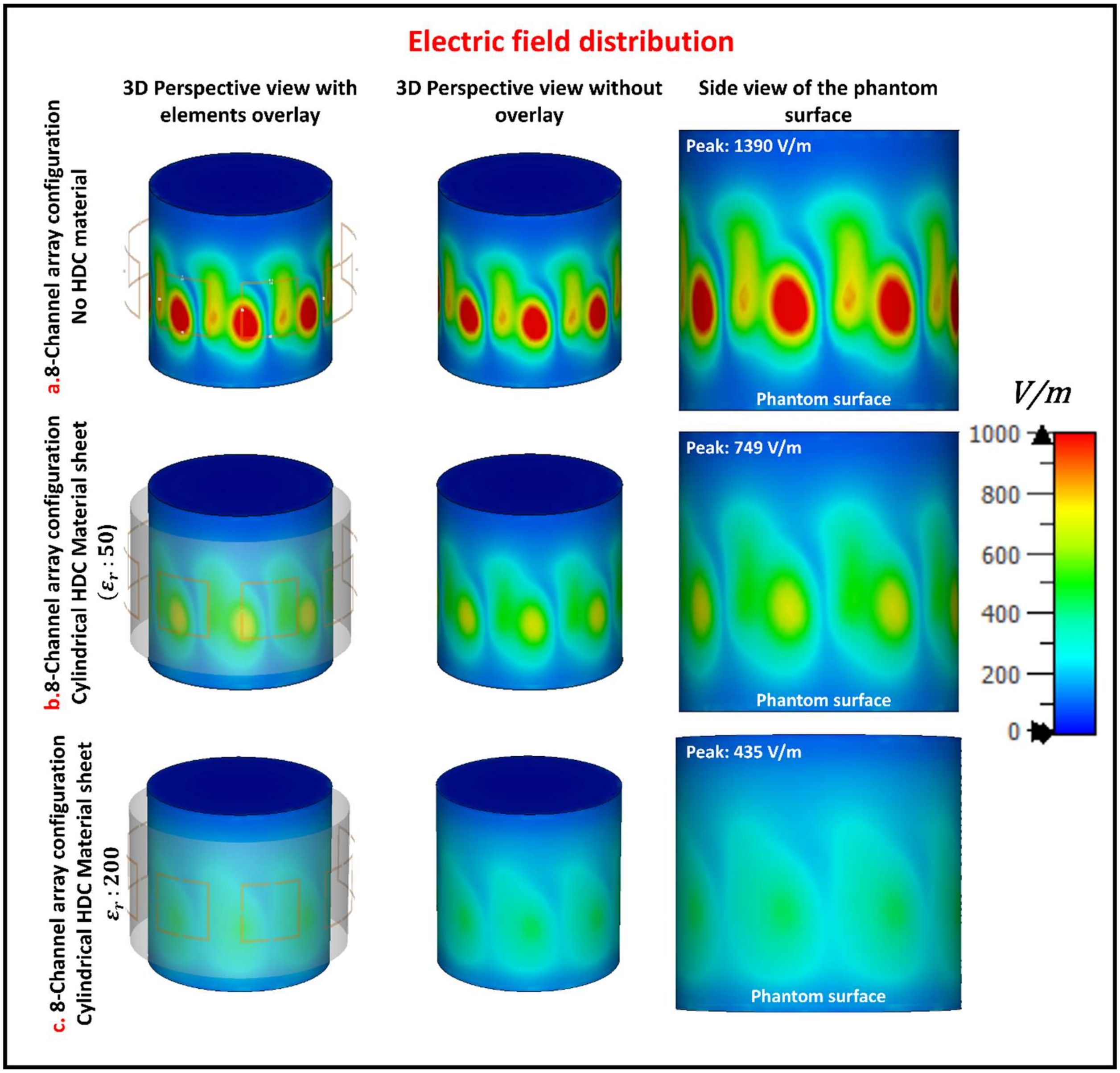

Fig 5. Electric field distribution on the phantom surface for the array configurations depicted in three views: 3D perspective with an overlay of coils and High-Dielectric Constant (HDC) material, 3D perspective without overlay, and a side profile illustrating the SAR fields on the phantom surface.
